# Not everywhere, not always: 30 targeted PFAS in ambient surface waters of Queensland, Australia

**DOI:** 10.1007/s10661-026-16030-5

**Published:** 2026-10-20

**Authors:** Brenda Baddiley, Albert Myburgh, Suzanne Vardy

**Affiliations:** Department of Environment, Tourism, Science and Innovation, Water Quality Monitoring and Investigations, Water Ecosystem Science, Ecosciences Precinct, Yuggera, Dutton Park, GPO Box 2454, Brisbane, QLD 4001 Australia

**Keywords:** PFAS, Ambient concentration, Sources, Surface waters, Urban, Remote

## Abstract

This study provides a summary of ambient concentrations of 30 commonly analysed perfluoroalkyl substances (PFAS) at 54 sites adjacent to various land use types across the east coast of Queensland, Australia. Sites were sampled for surface water every 2 months for a year (2019–2020). The results showed higher concentrations of PFAS in surface waters adjacent to major city areas (e.g. urban, industrial, residential land use types), and the lowest in surface waters adjacent to remote and regional areas (e.g. national parks, agriculture land use types). Perfluorooctanesulfonic acid (PFOS) was the most reported compound (in 53% of samples), ranging from < 0.1 to 0.4 ng L^−1^ in remote areas, < 0.1 to 0.6 ng L^−1^ in outer regional areas, < 0.1 to 4.7 ng L^−1^ in inner regional areas, and < 0.1 to 37.0 ng L^−1^ in major cities. No PFAS were reported at nine of the 54 sites during the six monitoring rounds, and 16 out of 30 PFAS were not reported at any of the sites sampled over the year. Although PFAS (such as PFOS) are described as ubiquitous, the results from this ambient monitoring program indicate that this assumption does not necessarily apply to all ambient surface waters in Queensland.

**Supplementary information:**

The online version contains supplementary material available at https://doi.org/10.1007/s10661-026-16030-5.

## Introduction

Per- and poly-fluoroalkyl substances (PFAS) are considered to be ubiquitous and are colloquially referred to as forever chemicals. PFAS have been detected in remote areas, from the Tibetan Plateau (Chen et al., 2021; Zhou et al., 2022) to polar ice caps (Ali et al., 2021; Shan et al., 2021). Although PFAS have been detected in remote areas (Cai et al., 2012), numerous studies have demonstrated a positive correlation between PFAS concentrations and population or major cities (Lee et al., 2020; Paige et al., 2024; Rankin et al., 2016; Sardiña et al., 2019, 2024; Scott et al., 2009), as well as related infrastructure such as wastewater treatment plants (WWTP), airports and defence bases, industries, and landfills (Ahrens, 2011; Evich et al., 2022; Jarvis et al., 2021; Loos et al., 2009; Zhang et al., 2016). Sample sites are often categorised by human activities or land use factors (Nguyen et al., 2022; Roscales et al., 2022; Sardiña et al., 2024; Tscharke et al., 2019), as larger urban areas and local industrial point sources are significant contributors to PFAS contamination. Agricultural watersheds may also represent potential diffuse sources of PFAS, such as some pesticides and biosolids that are applied to land (Peter & Lee, 2025).

In the Southern Hemisphere, the presence of PFAS in waters is reportedly lower than in the global north (Miranda et al., 2021; Savvidou et al., 2023; Shan et al., 2021; Yamashita et al., 2008) where PFAS production has historically been concentrated (Tansel, 2024). The World Population Review (Gaigbe-Togbe et al., 2022) lists the Southern Hemisphere as accounting for around 10% of the world’s population, with Australia itself only accounting for less than 0.5%. The average population density of the globe is 61 people per square kilometre, and Australia is one of the least densely populated areas of the world with only 3 people per square kilometre (Gaigbe-Togbe et al., 2022).

In Australia, PFAS have not been manufactured (Hagen et al., 2024), and only relatively small amounts are imported for direct use (Coggan et al., 2019), with firefighting activities and industrial applications considered significant sources in the environment (Seow, 2013). Other major contributors include the discharge of treated and untreated effluent into waterways (Gallen et al., 2022; Thompson et al., 2011a, 2011b), site contamination of groundwater (Gallen et al., 2017; Hepburn et al., 2019), and stormwater runoff (Álvarez-Ruiz et al., 2021; Pramanik et al., 2020). The majority of studies have historically focused around areas where point sources were located, or PFAS contamination has occurred. Therefore, data on ambient concentrations have been limited (Jarvis et al., 2021). This has encouraged the perception that all PFAS are ubiquitous which can lead to a reluctance to address contamination issues unless severe.

A number of PFAS studies including ambient monitoring data have been undertaken in Australia (Environment Protection Authority Tasmania, 2022; Paige et al., 2024; Richmond, 2022; Richmond & Nice, 2026; Sardiña et al., 2019, 2024). These vary across land use types, catchments, type of fresh and estuarine/coastal systems, number of sites, and number of samples collected, and generally these studies include one or two rounds of sampling.

Sardiña et al. (2019) investigated PFAS in surface waters at 25 freshwater sites (single round), across a number of land use in Victoria. They found PFHxS, PFOS, and PFOA were the most frequently detected overall (> 50%), with the highest concentrations found at residential and industrial sites. PFBA was the most commonly detected PFAS at areas defined as background sites. Paige et al. (2024) sampled surface waters at 65 freshwater sites in a single round around Melbourne, Victoria, with the majority of these being in urban areas. Twenty-two out of 33 targeted PFAS were reported, with PFOS being the most commonly detected (98% of samples), and with higher mean concentrations of all PFAS in urban areas compared to rural areas. Interestingly, PFBA was the compound with the highest mean concentration at rural sites. A further study of 85 Victorian inland freshwaters sites (one round) (Sardiña et al., 2024) across a range of different surrounding land uses found a distinct difference in ambient PFAS concentrations between urban and remote areas. PFAS were detected in 91% of water samples and 94% of these reported more than one PFAS. The sites where PFAS were not detected were all in remote areas.

The Government of Western Australia (WA) undertook two ambient studies with one being in 42 lakes in the Perth Metropolitan area over two seasons (Richmond, 2022) and one in 131 freshwater rivers and estuaries of southwest WA over one round (Richmond & Nice, 2026). Richmond (2022) reported detections of PFOS, PFHxS, and PFOA at all sites and both seasons except for two samples, with higher concentrations in lakes closer to the city. Richmond and Nice (2026) reported detections of PFOS at 52% of freshwater sites and 61% of estuarine sites with higher concentrations in areas with urban development. Nine other PFAS were detected; however, most were close to the LOR and reported at only a few sites.

The Tasmanian PFAS ambient monitoring program (Environment Protection Authority Tasmania 2022) looked at 76 freshwater and estuarine sites over two seasons, with PFOS being detected at 54% of sites and PFBA at 50% of sites. PFAS were not reported above the LOR at seven sites, and concentrations were higher in areas with more intense land use.

Data are limited on temporal PFAS concentrations in ambient waters in the Southern Hemisphere (particularly away from contaminated sites or point sources). Given the variation in PFAS concentrations and compounds reported around Australia, understanding the PFAS landscape in Queensland requires ambient data that are not specifically linked to areas impacted by point sources. The objective of this study was to provide a comprehensive dataset of the most commonly analysed PFAS concentrations in ambient surface waters across Queensland and to identify the primary environmental and landscape factors influencing their spatial and temporal variability.

## Materials and methods

### Sampling locations

A total of 54 sampling locations across the eastern coast of Queensland, Australia, were chosen (Fig. S1, Table S1) based on three criteria:

1. Sites were established monitoring sites within the Queensland Department of Environment, Tourism, Science and Innovation’s long-term monitoring program.
2. Sites were at least 1 km from WWTP and other known point sources of PFAS as recommended in the Stockholm Convention Guidance on the Global Monitoring Plan for Persistent Organic Pollutants (Stockholm Convention, 2015).
3. Sites were selected to represent different land use types/classes across the state. These were based on Australian Land Use and Management classification (ABARES, 2016) and included waters adjacent to major cities/urban areas, agricultural/rural areas, remote/conservation areas, and coastal areas.

Under the Queensland Government’s *Environmental Protection (Water and Wetland Biodiversity)* Policy 2019, the 54 sites included six sites described as freshwater sites, one lake/reservoir site, 42 estuarine sites, and five coastal sites (Table S1).

The Australian Statistical Geography Standard (ASGS) Remoteness Structure according to the Australian Bureau of Statistics (ABS) (Australian Bureau of Statistics, 2021) was used to divide areas into five classes: major cities, inner regional, outer regional, remote, and very remote. One-kilometre grids from the Accessibility/Remoteness Index of Australia (ARIA) are used by the ABS for each Statistical Area Level 1 (SA1) to define the remoteness class, and they are classified based on distance from populated localities and access to services.

At each site, in situ water quality variables and surface water grab samples were collected bimonthly between May 2019 and March 2020 and water quality variables were obtained using a multiparameter YSI EXO sonde (pH, turbidity, electrical conductivity (EC), dissolved oxygen (DO), temperature, salinity) (Table S2). Water samples were taken just under the surface (20–30 cm water depth), and at estuarine sites, samples were collected on an outgoing tide. PFAS samples were collected using laboratory supplied containers, with PFAS sampling protocols provided by CRC Care (CRC Care, 2018a, 2018b; CRC Care, 2018a, 2018b) and by the Western Australian Department of Environment Regulation (2016). Following these protocols, field blanks and transport blanks were collected as well as duplicate samples for intra- and inter-lab quality control checks (DES, 2018).

### Sample analysis

PFAS sampling and analysis follow that described in Vardy et al. (2024). Water samples were analysed by Eurofins, Brisbane, with interlaboratory duplicates analysed by Australian Laboratory Services (ALS), Brisbane. These laboratories are accredited by the National Association of Testing Authorities (NATA) for the analysis of PFAS, and samples were analysed at the lowest limit of reporting (LOR) available. LORs for each compound analysed are available in Table S3 and quality control details are summarised in supplementary information.

### Spatial analysis

To determine the population distribution across different catchments in Queensland, Australia, data obtained through Google Earth Engine and spatial analysis using QGIS were combined (see supplementary information for full method detail).

Using the WWF HydroSHEDS (version 2) and Center for International Earth Science Information Network (CIESIN) Gridded Population of the World (GPWv4) datasets, accessed through Google Earth Engine, total population was calculated in sampled catchments at two resolutions (level 6 (≈8000 km^2^) and level 12 (≈150 km^2^)) (CIESIN 2018; Lehner et al., 2022). This effectively produced a representation of total population in both the general vicinity of each site, as well as each sites greater catchment. A normalised elevation was assigned to each sample point representing the relative position of each site within its respective catchment (based on Shuttle Radar Topography Mission elevation data).

Distance to the closest potential PFAS source was determined using locations from Environmentally Relevant Activity (ERAs) Environmental Authority licenses under the *Environmental Protection Act 1994* and the *Environmental Protection Regulation 2008*. The ERAs used for this assessment were related to WWTP and other waste management industries (e.g. landfills); however, factors such as size or operational capacity were not considered.

The number of waste management ERAs and WWTP ERAs were determined using licensed locations associated with sewage or waste treatment infrastructure in the WWF HydroSHEDS level 12 catchment corresponding to each site.

To get an estimate of rainfall at sampling sites before sample collection, the Climate Hazards Group InfraRed Precipitation with Station data (CHIRPS) daily rainfall dataset (Funk et al., 2015) was used. The dataset, with a resolution of approximately 5.5 km, was used to calculate the 30-day average rainfall preceding the sampling dates for each site. For each site, a custom Google Earth Engine function extracted the relevant timeframe, filtered the CHIRPS data, and calculated the average precipitation in the cell corresponding to the site location for the month preceding the sampling date.

Each water sample therefore had the following variables associated with it: *date*, *conductivity*, *salinity*,* turbidity*, *water temperature*,* dissolved oxygen*,* pH*, *distance to known PFAS source*, *site elevation*, *rainfall prior to sampling*, *remoteness class*, *Level 6 Normalised Elevation*, *Level 12 Normalised Elevation*, *Level 6 Catchment Size*,* Level 12 catchment size*, *population in level 12 catchment*, *population in level 6 catchment*, *number of waste ERAs in level 12 catchment*,and *Number of WWTP ERAs in level 12 catchment.*

### Statistical analysis and censored data

As the most reported PFAS, PFOS was used as a surrogate for PFAS during statistical analysis. PFOS had the lowest LOR (0.1 ng L^−1^) and was reported at all sites where other PFAS were reported. Of 321 PFOS observations, 151 (47%) were below the LOR and were treated as left censored. PFOS descriptive statistics were estimated using regression on order statistics (ROS) in NADA2 (v2.0.2) in R, with the LOR supplied as the censoring threshold. ROS was used for distributional summary statistics only; modelled censored values were not assigned to individual samples or sites.

A correlation matrix was constructed using Spearman’s rank correlation coefficients on all numerical variables in the dataset. Any PFOS results below the LOR were replaced with half the LOR (0.05 ng L^−1^) (ANZG, 2018) before computing the geometric mean of PFOS concentrations (due to PFOS data being log-normally distributed) (USEPA, 2006) and the arithmetic mean of all other variables per site, resulting in a final dataset of 54 observations (1 per site). The strongest associations remained statistically significant when censored values were substituted with either half the LOR or the LOR, and when site means were calculated using reported PFOS concentrations only.

Multivariable linear models were used to identify combinations of landscape (e.g. population, remoteness class), PFAS source, and environmental variables associated with site-level PFOS concentrations. Water quality variables were not included as these change between sampling events and were not available for every site. PFOS values below the LOR (0.1 ng L⁻^1^) were represented by half the LOR (0.05 ng L⁻^1^), site geometric means were calculated across monitoring rounds, and PFOS was log-transformed before modelling. Models were evaluated using all-subsets selection and ranked by Akaike information criterion corrected (AICc), with a maximum of five predictor terms per model. Landscape and PFAS source predictors were assessed using all 54 sites. Best-supported models were assessed using adjusted R^2^, confidence intervals, regression diagnostics, and variance inflation factors (VIF/GVIF), with Tukey-adjusted pairwise comparisons used for remoteness class where applicable.

### Temporal analysis

Seasonal patterns were explored with sites that had at least one PFAS reported in water samples from all months. Only six sites fulfilled these criteria and were in the South East Queensland (SEQ) area. They were Oxley Creek, Brisbane River (Indooroopilly), Brisbane River (Karana Downs), Brisbane River (Lytton), Caboolture River (Caboolture), Moreton Bay (Bramble Bay), and Tingalpa Creek. Rainfall data were obtained from the Bureau of Meteorology Station numbers 40458 (Tingalpa), 40211 (Oxley), and 40965 (Clontarf) (BoM, 2026).

## Results and discussion

PFOS was the most frequently reported PFAS, being reported in 53% of all samples and at 83% of sites (45 of the 54 sites, Table 1). The LOR for PFOS was an order of magnitude lower than other PFAS, which may have contributed to this higher detection frequency. At 10 of these 45 sites, PFOS was reported in less than 50% of the samples over all monitoring rounds and was reported at concentrations close to or at the LOR (0.1 ng L^−1^). PFOS was reported at all sites where other PFAS were reported. The PFAS with the next highest reporting frequency was PFHxA at 13% of all samples, followed by PFOA, PFHxS, and PFPeA with reporting frequencies of 11.5%, 11.2%, and 11.2% of samples, respectively (Table 1). No PFAS were reported at nine of the 54 sites during any monitoring period with these sites being located in inner and outer regional, and remote areas (Figs. 1 and 2).

**Table 1:**
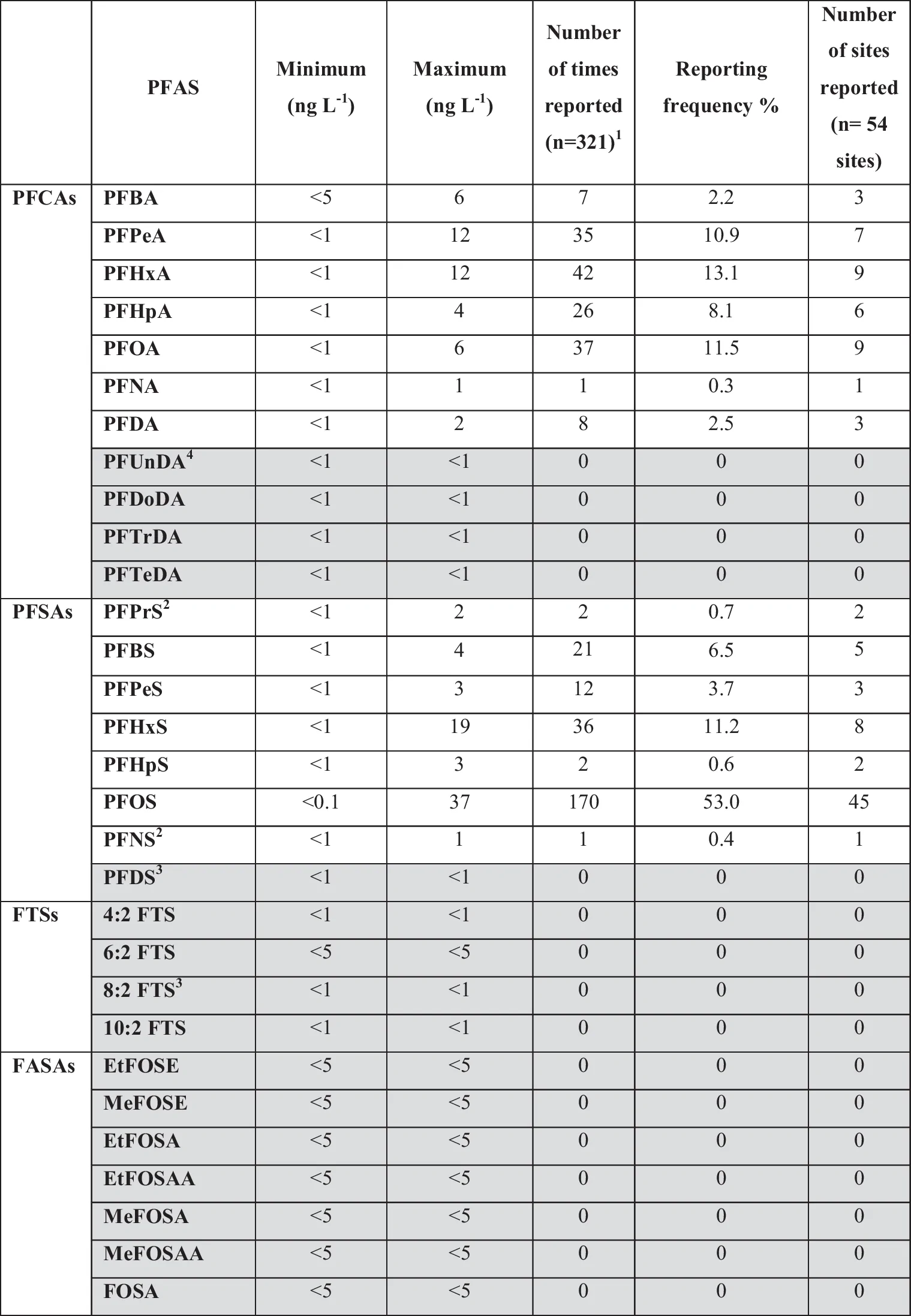
Summary of PFAS analysed in samples collected at all sites (*n* = 54) over six monitoring rounds, PFAS range, and reporting frequency. Grey-shaded cells are where the compound was not reported in any monitoring round at any site

^1^Three sites were not sampled during one round due to logistical reasons

^2^was not measured by the lab in the first round of sampling (*n* = 267)

^3^was reported in a single sample at a single site but not reported in any other samples or the duplicate of that sample

**Fig. 1:**
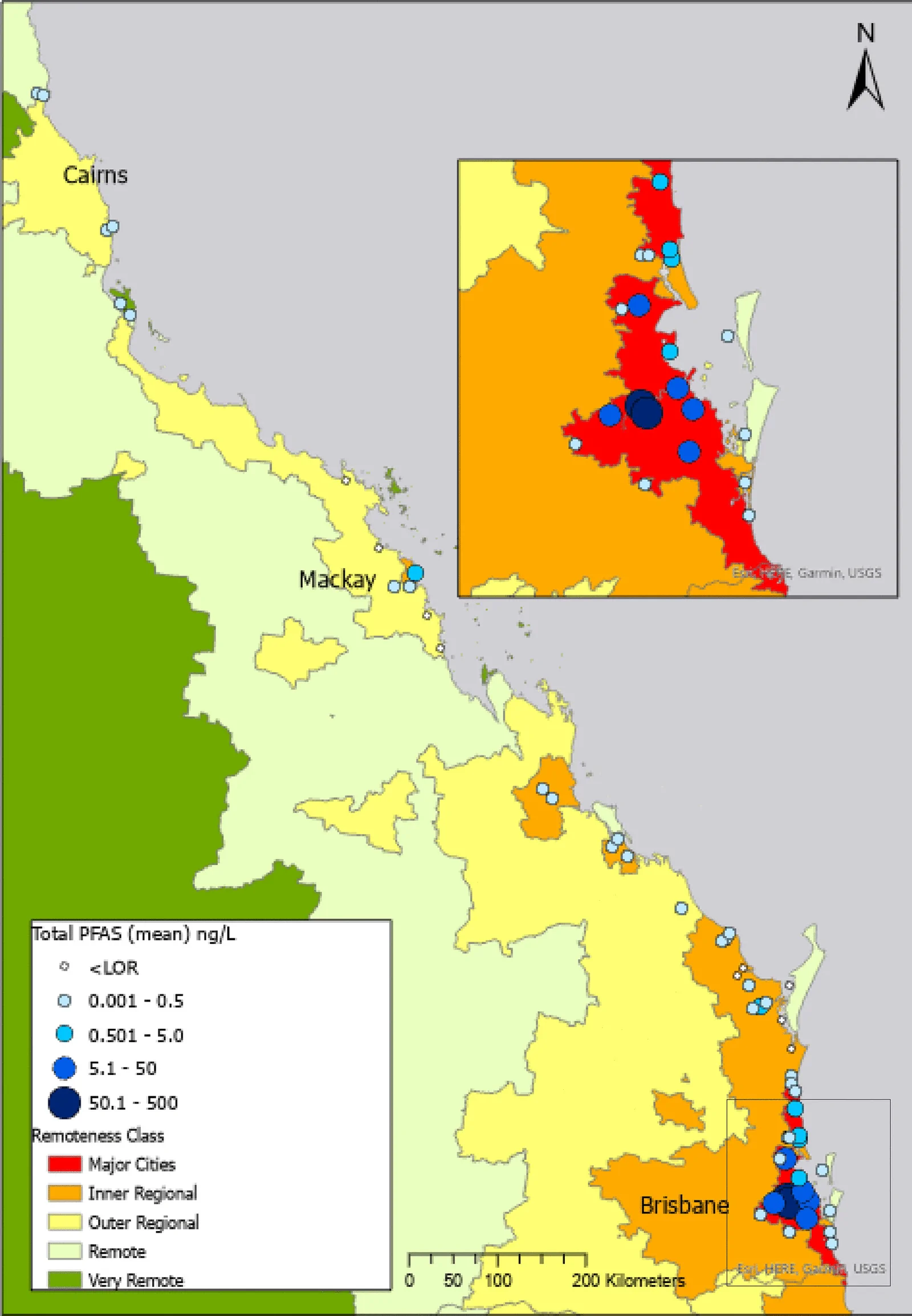
Mean total PFAS (ng L^−1^) in water for sites with 50% or more samples reported > LOR (Australian Bureau of Statistics, 2021; Esri Inc, 2021)

**Fig. 2:**
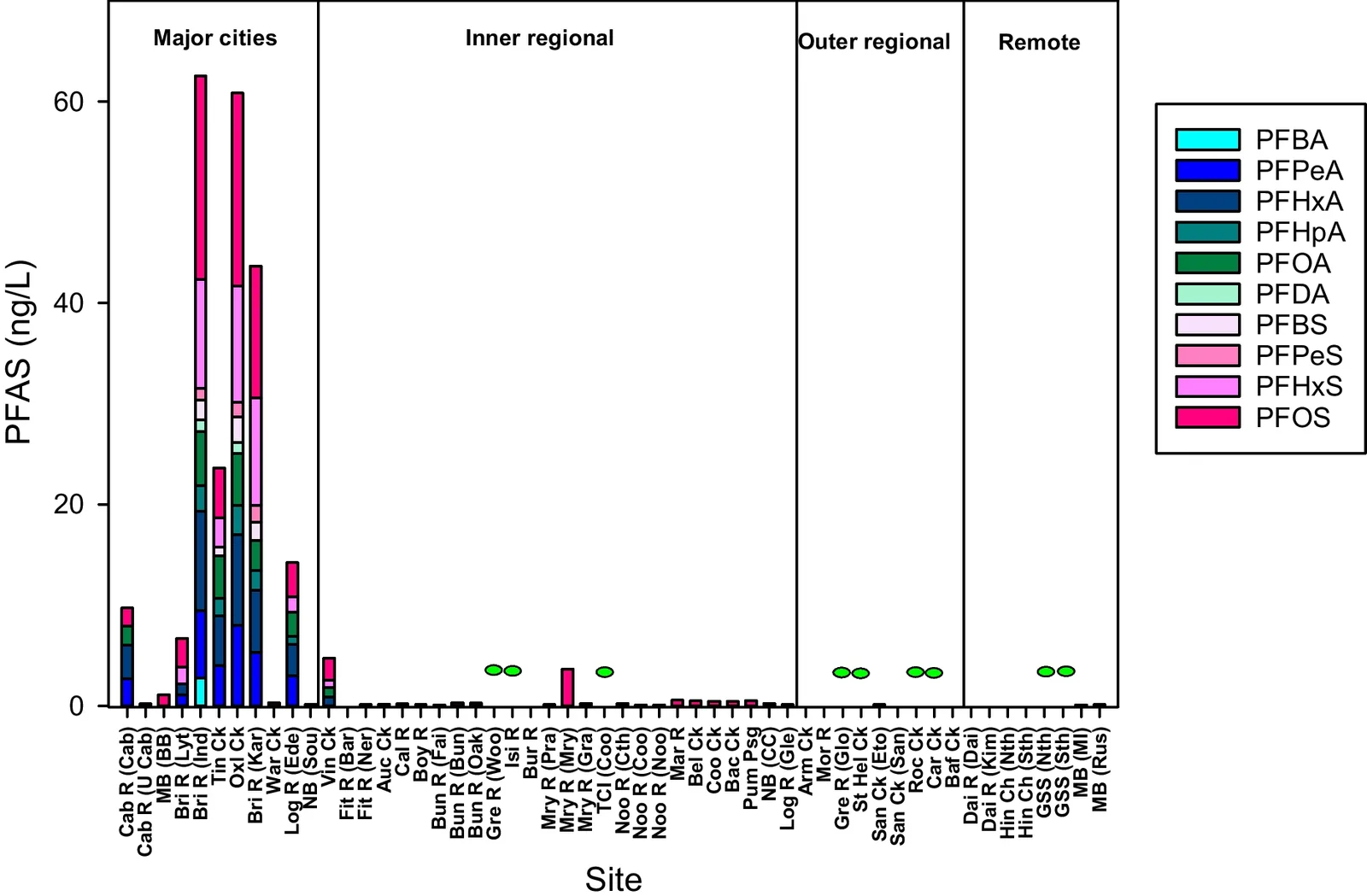
Mean concentrations of PFAS (ng L^−1^) at ambient sites in Queensland grouped by remoteness class. Sites with green dots are where no PFAS were reported over any monitoring round

Of the 30 compounds analysed, 16 PFAS were not reported above the LOR in any of the sampling rounds (Table 1). These compounds included four fluorotelomer sulfonic acids (FTS), seven perfluoroalkyl sulfonamides (FASA), one perfluoroalkyl sulfonic acid (PFSA), and four long-chain perfluoroalkyl carboxylic acids (PFCA).

Of the 54 sites sampled in this study, 11 fell into the major city class, 26 into inner regional, 9 into outer regional, and 8 into remote areas. None of the sites selected fell into the very remote class (Table S1). The mean concentrations of total PFAS (ng L^−1^) are shown by site and remoteness class in Fig. 1. The sites where PFAS was not measured in any sample were all in either the inner or outer regional remoteness class, and the highest concentrations were measured in the major city class.

Of the 54 sites that were sampled over a year, major cities (urban areas) accounted for the highest mean concentrations of total PFAS (up to 63 ng L^−1^) and had the highest number of PFAS reported (Fig. 2). PFOS was the only PFAS reported at 32 of the other 43 sites (Fig. 2), with the highest mean concentration of 3.6 ng L^−1^ at one site, although the majority of other sites were around the limit of reporting (0.1 ng L^−1^).

The nine sites where PFAS other than PFOS were reported were in the major city class, and all were in the more densely populated SEQ, apart from one site on the central coast in Queensland (Vines Creek, Mackay) which is in an inner regional area located adjacent to an industrial estate with a WWTP and landfill nearby (Fig. 2).

### Contributing factors to PFOS presence and concentrations

Site-level Spearman correlations with PFOS were strongest for level 12 catchment population (*ρ* = 0.71), distance to the nearest known PFAS source (*ρ* = − 0.66), and number of waste ERAs in the Level 12 catchment (*ρ* = 0.60) (Fig. 3). Their direction and significance were retained when censored observations were represented by the LOR, half LOR, and zero.

**Fig. 3:**
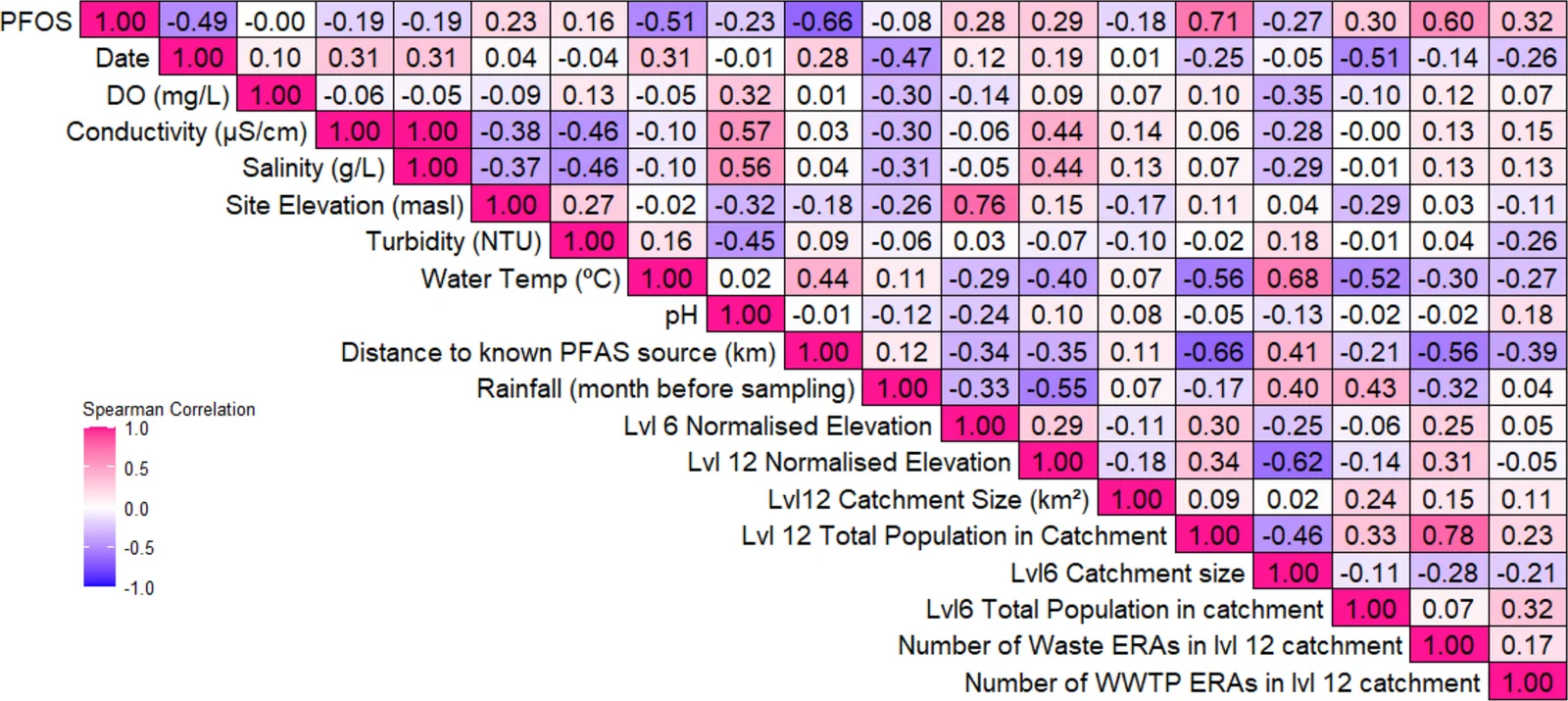
Correlation matrix indicating strong correlations with PFOS concentrations and population, distance to PFAS source, and number of waste ERAs in the catchment

The average size of a level 12 catchment basin is approximately 180 km^2^. More people are often associated with more infrastructure, and the number of waste ERAs tends to increase in catchments with greater population densities. The distance to the nearest potential PFAS source is affected by both the number of people and the number of waste ERAs since there is a higher likelihood of being closer to a waste ERA in more densely populated areas and PFOS concentrations tend to decrease with an increase in distance from a possible source.

Across all 54 sites, the best-supported multivariable linear model included remoteness class and level 12 population density (AICc = 154.77, adjusted *R*^*2*^ = 0.63). Results are available in the supplementary information. Collinearity was low, with adjusted GVIF values of 1.11 for remoteness class and 1.35 for population density.

Although distances from sampling sites to known PFAS sources were mapped, some waste activities can occur with minimal reporting or recording of information. Septic systems and irrigated wastewater can be a source of PFAS release to groundwater and local surface waters (Lenka et al., 2022; Schaider et al., 2016; Szabo et al., 2018). While atmospheric deposition has been attributed as a source in remote locations (Cai et al., 2012; Casal et al., 2017; Cousins et al., 2022; Evich et al., 2022; Podder et al., 2021; Szabo et al., 2022), it was noted by Thompson et al. (2011a, 2011b) that atmospheric precursors from the northern hemisphere were unlikely to affect areas of Australia, with local sources more likely to contribute to PFAS concentrations. The potential for atmospheric deposition has also led to the framing of PFAS as being ubiquitous in the environment; however, there have been no studies done (to the authors’ knowledge) to measure atmospheric deposition of PFAS in the Australian environment.

### PFAS in urban areas

In some urbanised sites near major cities (e.g. the Gold Coast), very low concentrations of PFAS (if any) were reported in water samples. This is likely associated with sampling sites being in coastal estuarine areas with very high water movement and flushing, and where potential sources such as sewage discharge points are located offshore (DETSI, 2016).

One of the sampling sites located at the mouth of the Brisbane River, a highly urbanised and industrialised river, and adjacent to one of Brisbane’s largest WWTP had lower concentrations of PFAS compared to the middle and upper Brisbane River sites (Fig. 4). Previous studies have shown that residence time of a pollutant or flushing time increases with increased distance up the Brisbane River (Álvarez-Ruiz et al., 2021; Dennison and Abal 1999; Gibbes et al., 2014). Earlier modelling of Moreton Bay, the coastal bay area adjacent to the Brisbane River (Dennison and Abal 1999; Gibbes et al., 2014), indicated flushing time is also further reduced in Moreton Bay where very low concentrations of PFAS have been reported in this study (Fig. 4). Gallen et al. (2014) also reported concentrations of PFAS along a spatial gradient of the Brisbane River out to Moreton Bay post flood which showed similar patterns.

**Fig. 4:**
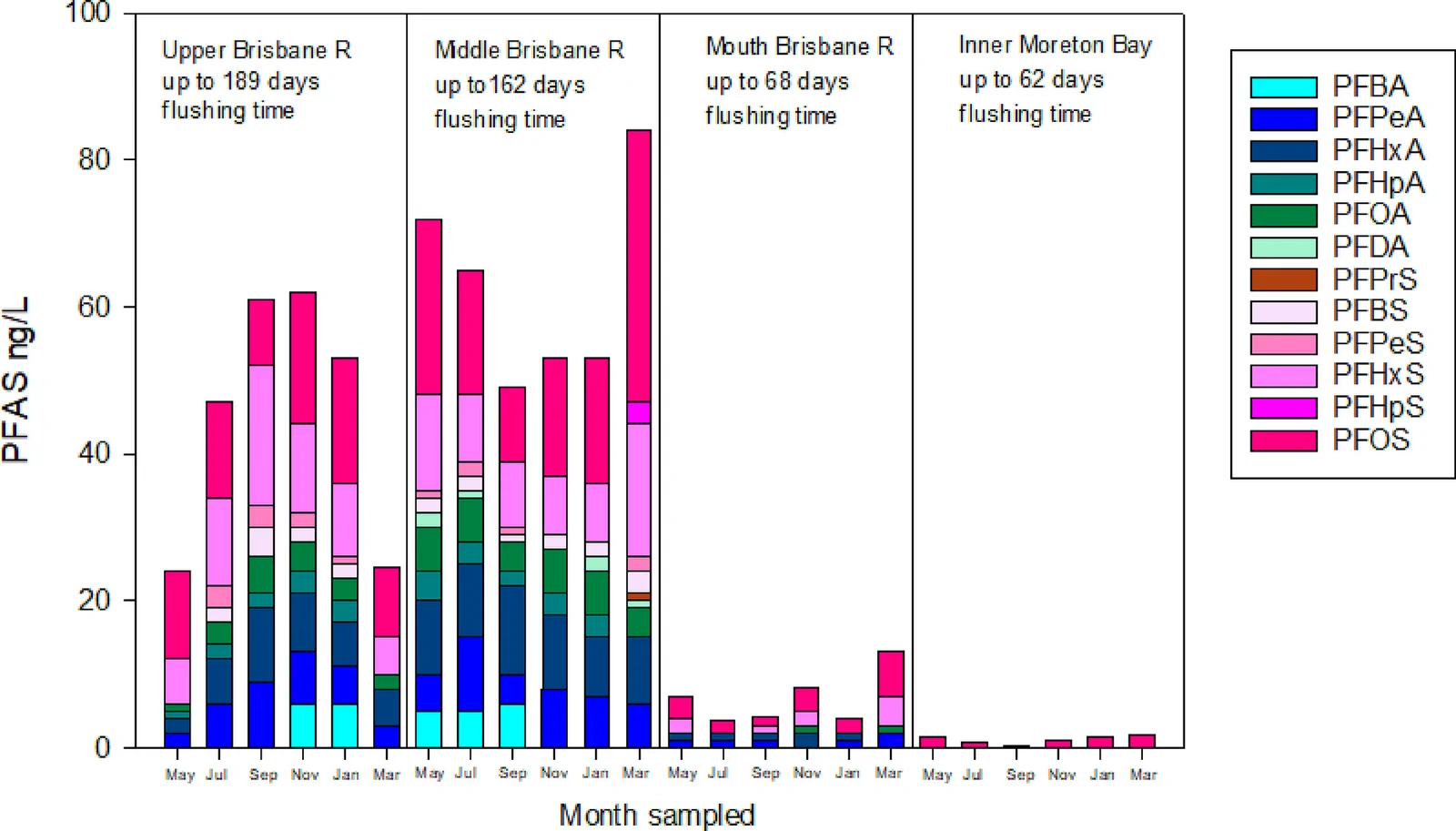
PFAS concentrations in the Brisbane River sites and adjacent Moreton Bay site in the major city class, with approximate water flushing times from Dennison and Abal (1999)

Ranges of reported concentrations of PFOS and PFHxS are similar across major cities and urban areas in Australia (Table 2).

**Table 2:** Concentration ranges of PFOS, PFOA, PFHxS, and PFBA found across urban areas in Australian studies

**State**	Queensland	Victoria	Victoria	WA	WA	Tasmania
Author, year	This study	Sardiña et al., 2024	Paige et al., 2024	Richmond, 2022	Richmond & Nice, 2026	EPA Tasmania, 2022
Water type^a^	E and FW	FW	FW	FW Lakes	FW, brackish	E and FW
Number of urban sites (monitoring rounds)	11 (6)	42(1)	51(1)	42 (2)	4(1)	12 (2)
Years sampled	2019–2020	2019	2020	2019	2020–2022	2020
PFOS (ng/L)	< 0.1–37	0.5–81	1.02–135	0.4–44	2.1–35	< 0.1–69
PFOA (ng/L)	< 1–6	0.5–36	1.6–34.5	< 1–380	1.0–2	< 0.1–25
PFHxS (ng/L)	< 1–19	0.5–44	0.4–38.5	< 1–80	< 1–43	< 0.2–20
PFBA (ng/L)	< 5–6	< 5–49	< 5–49.5	< 5–240	< 5–6	0.5–33

^a^E estuarine, FW freshwater

PFOA concentrations appear to be higher in most other states although the urban freshwater sites in WA (Richmond & Nice, 2026) report similar concentrations to Qld urban sites (Table 2).

PFBA was reported in only 2% of samples and only at two urban sites in this study with the range (up to 6 ng L^−^) contrasting with other Australian (and International) studies (Table 2). For example, Victoria reported PFBA in 98% of samples with concentrations ranging up to 49 ng L^−1^ (Sardiña et al., 2024), the Tasmanian study reported PFBA up to 33 ng L^−1^ (Environment Protection Authority Tasmania 2022), and the WA study found PFBA at many lake sites with a range up to 240 ng L^−1^ (Richmond, 2022). However, lakes can be potential sinks for PFAS, and at other WA urban freshwater sites, the reported concentrations were similar to the current study (Richmond & Nice, 2026). PFBA has also been reported in rural or remote sites in other areas of Australia (Paige et al., 2024; Sardiña et al., 2019). It is unclear why there are differences to the reported concentrations of PFOA and PFBA in this study, and this requires further investigation.

### PFOS concentrations and guidelines

The concentrations of PFOS were compared to the Australian and New Zealand Guidelines for Fresh and Marine Water Quality (ANZG, 2026). In Australia, most aquatic ecosystems are classified as “slightly to moderately disturbed” (ANZG, 2018). To protect these ecosystems, the recommended approach is to use a default guideline value (DGV) designed to safeguard 95% of species. For compounds that bioaccumulate and biomagnify, such as PFOS, the ANZG WQGs recommend using a more stringent DGV that protects 99% of species, which is 20 ng L^−1^ for PFOS. In addition, the ANZG guidelines (ANZG, 2026) include an interim screening threshold (IST) for PFOS (0.5 ng L^−1^). If a water concentration exceeds this threshold, sampling of aquatic organisms that may be consumed by higher order air-breathing animals (birds and mammals) is recommended to assess potential risks of bioaccumulation exceeding national guidelines protective of wildlife diet quality (HEPA, 2025). Across the 54 sites sampled in Queensland, only three sites (5%) had maximum concentrations above the DGV for PFOS (ANZG, 2026). They were Brisbane River at Indooroopilly (37 ng L^−1^), Oxley Creek (30 ng L^−1^), and Mary River at Maryborough (21 ng L^−1^) (Fig. 5a, b). The first two sites are linked, with the Brisbane River site being downstream of the discharge point from the Oxley Creek WWTP, and approximately 3 km upstream of the Oxley Creek confluence with the river. The elevated concentration at the Mary River site is an anomaly, as PFOS concentrations in all other monitoring rounds at this location were at or below the IST or below the limit of reporting (LOR). It is unclear what the cause of this result was (the duplicate returned a similar concentration so has been treated as a real result), and the source was not found at the time. Of the rest of the sampled sites, nine sites (16%) had no PFOS, and 26 sites (47%) had maximum concentrations of PFOS below the IST. This means that 63% of sites sampled in Queensland had concentrations of PFOS below those considered to be a risk based on the current guidance. Seventeen sites (31%) had maximum PFOS concentrations that exceeded the IST but were below the DGV, meaning these waters may pose a dietary risk to higher order air-breathing animals that live in and around these waters. Sites that exceeded the PFOS 99% DGV and/or IST were found in the major cities (Fig. 5a) and inner regional (Fig. 5b) remoteness classes. Neither outer regional (Fig. 5c) nor remote (Fig. 5d) classes had PFOS concentrations that exceeded the IST.

**Fig. 5:**
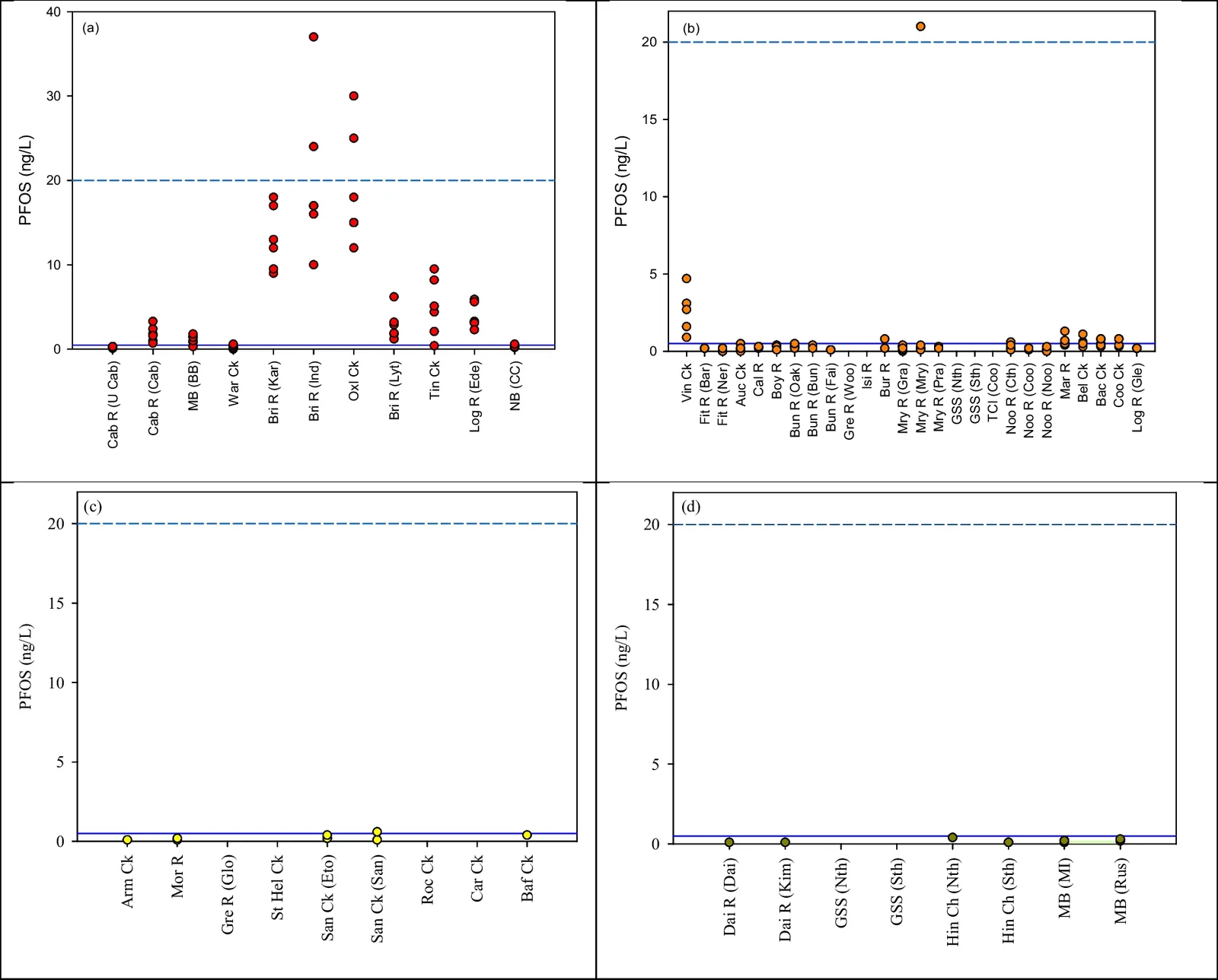
Bi-monthly PFOS concentrations in **a** major cities, **b** inner regional, **c** outer regional, and **d** remote site classes. Dashed blue line denotes the ANZG (2026) 99% DGV and blue line denotes the IST. Note: concentrations less than the limit of reporting were not graphed

### PFAS and temporal patterns

This study revealed different temporal patterns across sites in the study, either an increase of PFAS in the wet season, an increase of PFAS in the dry season, or no pattern. In this study, two sites had an increase of PFAS in the dry season—Brisbane River (Karana Downs) and Caboolture River (Caboolture) (Fig. 5a and Fig. S3a respectively). Both these sites are in tidal estuaries in the major city remoteness class and are about a kilometre upstream from licensed sewage discharge points. The Caboolture River site is downstream of the town of Caboolture, considered an outer suburb of Brisbane with rural land upstream. The Brisbane River (Karana Downs) is in the upper estuary and downstream of urban residential and peri-urban land, with headwaters in conservation areas. The flow in both rivers is seasonal, with the highest flow occurring in the wet season. Effluent from the downstream WWTP at both sites are discharged consistently and may be a potential source of PFAS accumulating in the dry season. In contrast, Oxley Creek and Brisbane River at Indooroopilly (Fig. 6b and Fig. S3b respectively) show elevated levels of PFAS in the wet season. As mentioned previously, these sites are considered to be linked. The Oxley Creek catchment is highly industrialised and has many potential sources of PFAS, such as a small airport, waste landfills, heavy industry, and resource recovery industries, as well as one of the largest WWTP in the Brisbane region. The increase in the wet season may be associated with stormwater runoff from the industrial areas as well as wet weather releases from the WWTP into Oxley Creek. Finally, there appears to be no seasonal pattern associated with the Tingalpa site (Fig. 6c), likely associated with the number of different sources upstream of the site in this small creek (e.g. a WWTP and an old landfill). Of note, an extra sample was taken opportunistically from Bramble Bay after heavy rains. This site is adjacent to the mouth of the Pine River, and the North Pine River is known to be contaminated with PFAS (Baddiley et al., 2020). The sample was taken during a period following intense rainfall and the total PFAS concentration was approximately six times higher than the other months, with PFPeA, PFHxA, PFOA and PFHxS being detected in addition to PFOS (Fig. 6d).

**Fig. 6:**
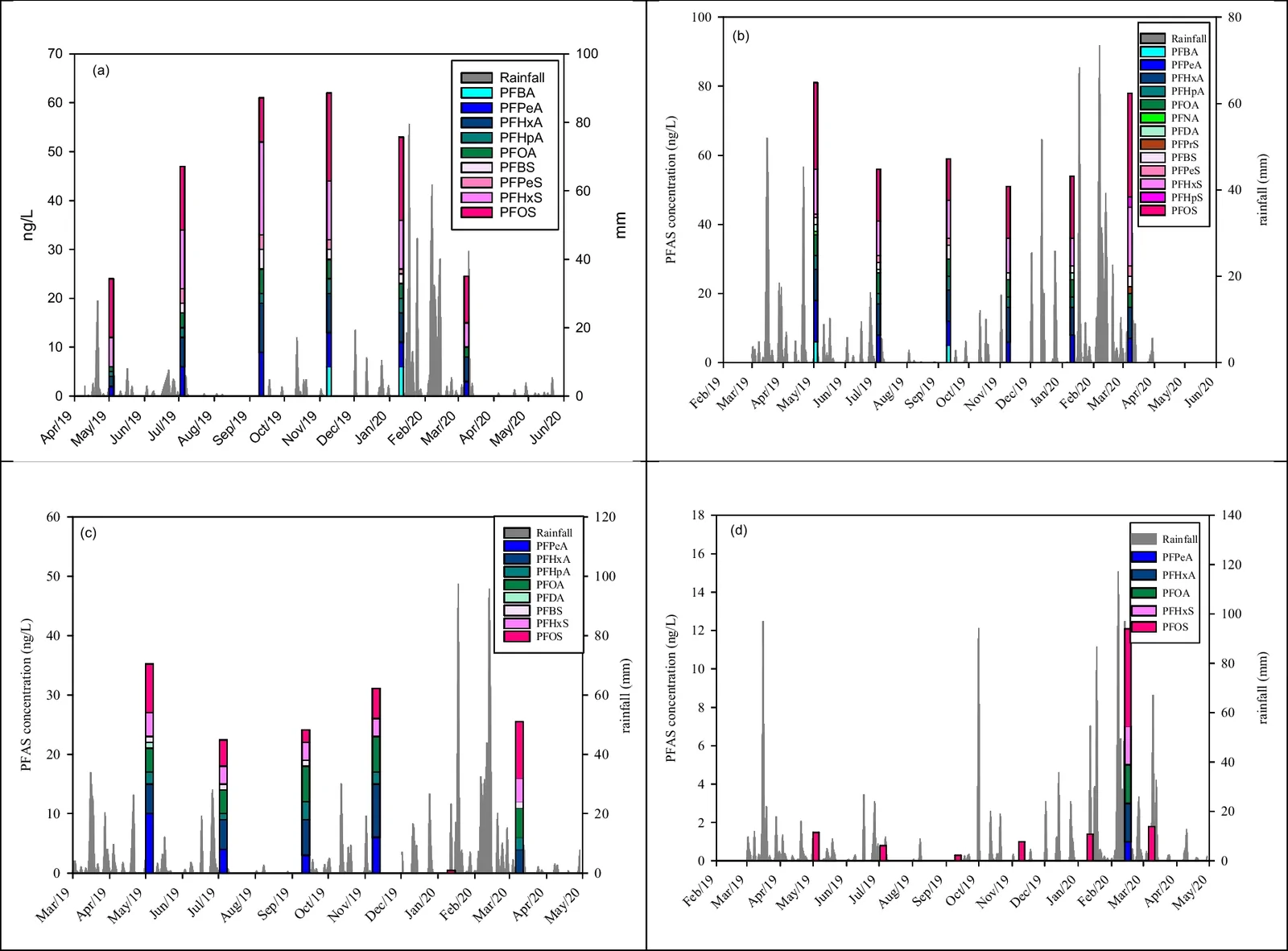
Seasonal patterns in total PFAS at **a** Brisbane River at Karana Downs, **b** Oxley Creek, **c** Tingalpa Creek, and **d** Bramble Bay

Collectively, these findings demonstrate that temporal variability in PFAS concentrations is strongly influenced by site-specific source characteristics and hydrological conditions. Given the limited number of monitored locations, the observed wet season and dry season patterns should be interpreted as site-specific responses rather than broader regional trends. Longer-term monitoring across a larger number of sites would be required to determine whether these patterns are representative of wider seasonal processes in Queensland surface waters. The results illustrate the importance of temporal coverage when assessing a site.

## Conclusions

The findings presented here challenge the assumption that commonly analysed PFAS (and specifically PFOS) are everywhere, as no analysed PFAS were reported above the LOR at nine of 54 ambient surface water sites across six monitoring rounds in Queensland. This was also found in another study of PFAS in the biota of South East Queensland freshwaters ( Vardy et al., 2025) which found that PFAS were not reported above the LOR in fish and crustaceans at nine of 31 sites.

PFOS concentrations were associated with population density and remoteness class, with higher concentrations in major cities/urban areas. Ambient concentrations of PFOA and PFBA were generally lower than those reported in other urban areas in Australia. The assumption of PFAS ubiquity from sources such as atmospheric deposition based on findings from the Northern Hemisphere may not always be applicable in the ambient surface waters of Queensland. This aligns with other reports suggesting that PFAS concentrations in the Southern Hemisphere are considerably lower than those in the Northern Hemisphere, where manufacturing and industrial use have historically been concentrated and population density is much higher.

In Queensland, data gaps exist in areas with septic systems, biosolid and pesticide applications, and recycled water irrigation, as well as potential contributions from atmospheric deposition. Ambiguities around long-range transport pathways further complicate understanding PFAS distribution, particularly in remote areas, and might be easily exploited and accepted as a reason why PFAS may be everywhere.

Expanding the geographic scope of sampling efforts, including in freshwater systems, will help build a more comprehensive understanding of PFAS distribution across Queensland. Additionally, more data on seasonal influences are required, as this study found that this is a very site-specific variable, and one that can be influenced by many other factors.

### Limitations

PFAS limits of reporting and methods for analyses within laboratories are constantly evolving; some of the LORs for this study were higher than those in current literature and since the monitoring was undertaken, and more PFAS are being described (and analysed). Sites were selected based on existing monitoring program so freshwater sites were limited as well as sites further inland from the coast.

## Supplementary information

Below is the link to the electronic supplementary material.

ESM 1 (DOCX 2.63 MB)

ESM 2 (CSV 23.0 KB)

## Data Availability

Publicly available at: https://www.qld.gov.au/environment/management/environmental/pfas/monitoring-program-report
