# Supplementary material for: Not everywhere, not always: 30 targeted PFAS in ambient surface waters of Queensland, Australia
Source: Environ Monit Assess. 2026 Oct 20;198(11):1232. doi: 10.1007/s10661-026-16030-5 (PMC13683488; doi:10.1007/s10661-026-16030-5)
Supplement: Supplementary file 1 [file 10661_2026_16030_MOESM1_ESM.docx]

**Supplementary Information**

**Not everywhere, not always: 30 targeted PFAS in ambient surface waters of Queensland, Australia**

**Brenda Baddiley^*^, Albert Myburgh, Suzanne Vardy**

Water Quality and Investigations, Water Ecosystem Science, Department of Environment, Tourism, Science, and Innovation.

^*^ Correspondence: Brenda Baddiley, Senior Scientist, Water Quality and Investigations, Water Ecosystem Science, Department of Environment, Tourism, Science, and Innovation. Email: brenda.baddiley@detsi.qld.gov.au

**Fig. S1** Sampling locations in Queensland

**Table S1** Sampling locations and site details

| Sampling location | Abbreviation | Latitude | Longitude | ^[1]^Water Type^[2]^ | Remoteness class^[3]^ |
| --- | --- | --- | --- | --- | --- |
| Armit Creek | Arm Ck | -17.6081 | 146.11089 | Middle estuary | Outer regional |
| Auckland Creek | Auc Ck | -23.8384 | 151.25065 | Lower estuary | Inner regional |
| Back Creek | Bac Ck | -26.8802 | 152.96739 | Lowland freshwaters | Inner regional |
| Baffle Creek | Baf Ck | -24.5423 | 151.90452 | Middle estuary | Outer regional |
| Bells Creek | Bel Ck | -26.8531 | 153.09281 | Middle estuary | Inner regional |
| Boyne River | Boy R | -24.0044 | 151.34098 | Middle estuary | Inner regional |
| Brisbane River (Indooroopilly) | Bri R (Ind) | -27.5058 | 152.97069 | Middle estuary | Major cities |
| Brisbane River (Karana Downs) | Bri R (Kar) | -27.5421 | 152.84143 | Upper estuary | Major cities |
| Brisbane River (Lytton) | Bri R (Lyt) | -27.4278 | 153.12607 | Middle estuary | Major cities |
| Burnett River (Bundaberg) | Bun R (Bun) | -24.8617 | 152.3564 | Middle estuary | Inner regional |
| Burnett River (Fairymead) | Bun R (Fai) | -24.7996 | 152.37811 | Middle estuary | Inner regional |
| Burnett River (Oakwood) | Bun R (Oak) | -24.8644 | 152.30405 | Middle estuary | Inner regional |
| Burrum River | Bur R | -25.3149 | 152.5893 | Middle estuary | Inner regional |
| Caboolture River (Caboolture) | Cab R (Cab) | -27.0868 | 152.95863 | Upper estuary | Major cities |
| Caboolture River (Upper Caboolture) | Cab R (U Cab) | -27.0979 | 152.8911 | Lowland freshwaters | Major cities |
| Calliope River | Cal R | -23.9137 | 151.183 | Middle estuary | Inner regional |
| Carmila Creek | Car Ck | -21.899 | 149.45466 | Middle estuary | Outer regional |
| Coochin Creek | Coo Ck | -26.8771 | 153.0034 | Lowland freshwaters | Inner regional |
| Daintree River (Daintree) | Dai R (Dai) | -16.2592 | 145.39806 | Middle estuary | Remote |
| Daintree River (Kimberley) | Dai R (Kim) | -16.2517 | 145.34806 | Middle estuary | Remote |
| Fitzroy River (Barrage) | Fit R (Bar) | -23.3168 | 150.47934 | Lakes/reservoirs | Inner regional |
| Fitzroy River (Nerimbera) | Fit R (Ner) | -23.4209 | 150.58995 | Middle estuary | Inner regional |
| Great Sandy Straits (Northern End) | GSS (Nth) | -25.3214 | 153.00262 | Lower estuary | Remote |
| Great Sandy Straits (Southern End) | GSS (Sth) | -25.6867 | 152.92455 | Lower estuary | Remote |
| Gregory River (Cape Gloucester) | Gre R (Glo) | -20.1804 | 148.48233 | Middle estuary | Outer regional |
| Gregory River (Woodgate) | Gre R (Woo) | -25.1483 | 152.51861 | Middle estuary | Inner regional |
| Hinchinbrook Channel (Northern End) | Hin Ch (Nth) | -18.3856 | 146.19694 | Enclosed coastal waters | Remote |
| Hinchinbrook Channel (Southern End) | Hin Ch (Sth) | -18.4942 | 146.28889 | Enclosed coastal waters | Remote |
| Isis River | Isi R | -25.2192 | 152.46494 | Middle estuary | Inner regional |
| Logan River (Eden’s Landing) | Log R (Ede) | -27.6954 | 153.16886 | Middle estuary | Major cities |
| Logan River (Glenlogan) | Log R (Gle) | -27.8336 | 152.98778 | Lowland freshwaters | Inner regional |
| Maroochy River | Mar R | -26.5702 | 153.05042 | Middle estuary | Inner regional |
| Mary River (Grahams Creek) | Mry R (Gra) | -25.5633 | 152.62437 | Middle estuary | Inner regional |
| Mary River (Maryborough) | Mry R (Mry) | -25.5388 | 152.7074 | Middle estuary | Inner regional |
| Mary River (Prawle) | Mry R (Pra) | -25.5018 | 152.7524 | Middle estuary | Inner regional |
| Moresby River | Mor R | -17.6382 | 146.06025 | Middle estuary | Outer regional |
| Moreton Bay (Bramble Bay) | MB (BB) | -27.2784 | 153.08771 | Enclosed coastal | Major cities |
| Moreton Bay (N of Russell Is) | MB (Rus) | -27.6247 | 153.4033 | Enclosed coastal | Remote |
| Moreton Bay (Near Moreton Is) | MB (MI) | -27.2125 | 153.33308 | Open coastal | Remote |
| Noosa River (Cooroibah) | Noo R (Coo) | -26.3215 | 153.02003 | Upper estuary | Inner regional |
| Noosa River (Lake Cootharaba) | Noo R (Cth) | -26.2406 | 153.0231 | Upper estuary | Inner regional |
| Noosa River Noosaville | Noo R (Noo) | -26.396 | 153.05557 | Lower estuary | Inner regional |
| Northern Broadwater (Couran Cove) | NB (CC) | -27.8246 | 153.40738 | Lower estuary | Inner regional |
| Northern Broadwater (Southport) | NB (Sou) | -27.9597 | 153.4211 | Lower estuary | Major cities |
| Oxley Creek | Oxl Ck | -27.5355 | 152.99189 | Middle estuary | Major cities |
| Pumicestone Passage | Pum Psg | -26.8955 | 153.09866 | Middle estuary | Inner regional |
| Rocky Dam Creek | Roc Ck | -21.5628 | 149.31455 | Middle estuary | Outer regional |
| Sandy Creek (Eton) | San Ck (Eto) | -21.262 | 148.97667 | Fresh waters | Outer regional |
| Sandy Creek (Sandiford) | San Ck (San) | -21.2712 | 149.1266 | Middle estuary | Outer regional |
| St Helens Creek | St Hel Ck | -20.8803 | 148.82265 | Middle estuary | Outer regional |
| Tin Can Inlet (Cooloola) | TCI (Coo) | -25.9783 | 153.01157 | Lower estuary | Inner regional |
| Tingalpa Creek | Tin Ck | -27.5194 | 153.18698 | Middle estuary | Major cities |
| Vines Creek (Mackay) | Vin Ck | -21.12 | 149.19736 | Middle estuary | Inner regional |
| Warrill Creek | War Ck | -27.6635 | 152.7 | Lowland freshwaters | Major cities |

**Table S2** In situ water quality variables taken at each site for each monitoring round

| **Sampling location** | **Date Time** | **Monitoring round** | **Temperature (ºC)** | **Dissolved Oxygen (mg/L)** | **Conductivity (µS/cm)** | **pH** | **Salinity (g/L)** | **Turbidity (NTU)** |
| --- | --- | --- | --- | --- | --- | --- | --- | --- |
| **Daintree River (Kimberley)** | 30/04/2019 11:04:00 | May 2019 | 24.8 | 7.21 | 131 | 7.86 | 0.06 | 5.6 |
| **Daintree River (Kimberley)** | 2/07/2019 10:42:56 | July 2019 | 21.96 | 7.87 | 3020 | 8.59 | 1.58 | 5.7 |
| **Daintree River (Kimberley)** | 10/09/2019 10:36:11 | September 2019 | 23.45 | 7.43 | 16540 | 8.08 | 9.71 | 3.6 |
| **Daintree River (Kimberley)** | 12/11/2019 11:58:12 | November 2019 | 28.94 | 6.27 | 25030 | 7.71 | 15.16 | 2.7 |
| **Daintree River (Kimberley)** | 21/01/2020 11:51:21 | January 2020 | 31.47 | 5.68 | 22090 | 7.53 | 13.18 | 3.7 |
| **Daintree River (Kimberley)** | 19/03/2020 10:14:55 | March 2020 | 27.24 | 7.02 | 6010 | 8.33 | 3.25 | 4.1 |
| **Daintree River (Daintree)** | 30/04/2019 12:24:00 | May 2019 | 24.1 | 7.36 | 76 | 7.37 | 0.03 | 5.7 |
| **Daintree River (Daintree)** | 2/07/2019 12:13:03 | July 2019 | 21.39 | 8.32 | 140 | 8.91 | 0.07 | 5.2 |
| **Daintree River (Daintree)** | 10/09/2019 11:39:04 | September 2019 | 22.45 | 8.09 | 600 | 9.22 | 0.29 | 4 |
| **Daintree River (Daintree)** | 12/11/2019 13:05:18 | November 2019 | 28.73 | 7.07 | 2340 | 7.28 | 1.12 | 3.2 |
| **Daintree River (Daintree)** | 21/01/2020 12:44:08 | January 2020 | 31.3 | 6.15 | 1440 | 8.41 | 0.71 | 4.4 |
| **Daintree River (Daintree)** | 19/03/2020 11:00:31 | March 2020 | 26.43 | 6.62 | 120 | 7.83 | 0.06 | 3.1 |
| **Moresby River** | 1/05/2019 11:10:00 | May 2019 | 24.43 | 5.37 | 703 | 6.68 | 0.34 | 15.7 |
| **Moresby River** | 3/07/2019 13:13:22 | July 2019 | 21.3 | 6.74 | 8680 | 8 | 4.85 | 5 |
| **Moresby River** | 11/09/2019 10:23:32 | September 2019 | 21.97 | 7.23 | 32740 | 7.66 | 21.9 | 2.9 |
| **Moresby River** | 13/11/2019 10:52:06 | November 2019 | 28.13 | 4.97 | 42480 | 7.33 | 27.21 | 1.8 |
| **Moresby River** | 22/01/2020 10:38:08 | January 2020 | 31.24 | 3.66 | 47210 | 7.08 | 30.52 | 2.6 |
| **Moresby River** | 17/03/2020 10:14:44 | March 2020 | 26.71 | 3.62 | 16100 | 7.77 | 9.4 | 5.1 |
| **Armit Creek** | 1/05/2019 10:06:00 | May 2019 | 26.1 | 5.86 | 45000 | 8.02 | 29.07 | 10.3 |
| **Armit Creek** | 3/07/2019 11:46:09 | July 2019 | 21.62 | 6.93 | 43430 | 8.03 | 28.03 | 5.4 |
| **Armit Creek** | 11/09/2019 8:51:39 | September 2019 | 22.29 | 8.33 | 49100 | 8.32 | 34.1 | 2.6 |
| **Armit Creek** | 13/11/2019 9:28:54 | November 2019 | 27.42 | 6.64 | 49240 | 8.28 | 32.12 | 0.6 |
| **Armit Creek** | 22/01/2020 9:02:29 | January 2020 | 29.36 | 6.14 | 52350 | 8.01 | 34.35 | 1.5 |
| **Armit Creek** | 17/03/2020 8:54:02 | March 2020 | 28.68 | 5.52 | 47950 | 8.18 | 31.14 | 5.6 |
| **Hinchinbrook Channel (Northern end)** | 2/05/2019 9:37:00 | May 2019 | 25.98 | 5.83 | 40360 | 7.88 | 25.75 | 1.3 |
| **Hinchinbrook Channel (Northern end)** | 4/07/2019 12:26:45 | July 2019 | 22.03 | 6.67 | 42760 | 8.13 | 27.54 | 3.2 |
| **Hinchinbrook Channel (Northern end)** | 12/09/2019 9:32:02 | September 2019 | 22.79 | 6.93 | 47390 | 7.95 | 32.4 | 3.5 |
| **Hinchinbrook Channel (Northern end)** | 14/11/2019 11:09:42 | November 2019 | 29.59 | 5.91 | 51130 | 7.77 | 33.44 | 1.1 |
| **Hinchinbrook Channel (Northern end)** | 23/01/2020 10:50:15 | January 2020 | 31.21 | 5.51 | 54160 | 7.84 | 35.62 | 3.1 |
| **Hinchinbrook Channel (Northern end)** | 18/03/2020 8:43:33 | March 2020 | 28.3 | 5.44 | 41860 | 8.13 | 26.76 | 0.9 |
| **Hinchinbrook Channel (Southern end)** | 2/05/2019 8:32:00 | May 2019 | 25.75 | 6.43 | 48780 | 8.12 | 31.83 | 17.4 |
| **Hinchinbrook Channel (Southern end)** | 4/07/2019 11:27:00 | July 2019 | 22.19 | 7.03 | 42170 | 8.25 | 27.11 | 5.8 |
| **Hinchinbrook Channel (Southern end)** | 12/09/2019 8:30:46 | September 2019 | 22.12 | 7.02 | 48870 | 8.33 | 31.96 | 8.3 |
| **Hinchinbrook Channel (Southern end)** | 14/11/2019 12:12:15 | November 2019 | 28.4 | 6.43 | 50590 | 8.13 | 33.08 | 2.9 |
| **Hinchinbrook Channel (Southern end)** | 23/01/2020 9:52:04 | January 2020 | 29.98 | 6.04 | 51610 | 7.92 | 33.78 | 5.8 |
| **Hinchinbrook Channel (Southern end)** | 18/03/2020 7:55:52 | March 2020 | 28.13 | 6.11 | 48150 | 8.37 | 31.3 | 4.5 |
| **Gregory River (Cape Gloucester)** | 14/05/2019 14:31:48 | May 2019 | 23.1 | 6.67 | 25260 | 7.51 | 15.4 | 10.4 |
| **Gregory River (Cape Gloucester)** | 25/07/2019 11:25:50 | July 2019 | 21.33 | 7.08 | 18850 | 7.53 | 11.21 | 3.3 |
| **Gregory River (Cape Gloucester)** | 26/09/2019 13:10:06 | September 2019 | 25.79 | 5.79 | 53020 | 7.67 | 34.95 | 7 |
| **Gregory River (Cape Gloucester)** | 19/11/2019 9:52:41 | November 2019 | 29.59 | 4.41 | 59140 | 7.5 | 39.42 | 2.17 |
| **Gregory River (Cape Gloucester)** | 16/01/2020 10:18:37 | January 2020 | 30.71 | 4.04 | 35740 | 7.25 | 22.4 | 1.82 |
| **Gregory River (Cape Gloucester)** | 17/03/2020 13:14:58 | March 2020 | 28.19 | 5.71 | 3280 | 7.19 | 1.7 | 14.15 |
| **St Helens Creek** | 15/05/2019 15:14:43 | May 2019 | 22.77 | 8.09 | 9410 | 7.43 | 5.28 | 16.7 |
| **St Helens Creek** | 23/07/2019 10:14:52 | July 2019 | 19.99 | 8.37 | 10930 | 7.47 | 6.22 | 10.8 |
| **St Helens Creek** | 24/09/2019 12:25:45 | September 2019 | 26.2 | 6.73 | 40490 | 7.6 | 25.84 | 9.9 |
| **St Helens Creek** | 20/11/2019 12:51:24 | November 2019 | 32.13 | 6.25 | 48890 | 7.24 | 31.71 | 8.37 |
| **St Helens Creek** | 14/01/2020 8:18:50 | January 2020 | 28.29 | 4.42 | 27180 | 6.99 | 16.6 | 8.45 |
| **St Helens Creek** | 18/03/2020 13:31:55 | March 2020 | 27.06 | 8.21 | 2850 | 7.34 | 1.47 | 9.77 |
| **Vines Creek** | 15/05/2019 16:56:57 | May 2019 | 23.26 | 5.74 | 18390 | 7.14 | 10.9 | 13.2 |
| **Vines Creek** | 22/07/2019 17:22:38 | July 2019 | 22.36 | 6.81 | 31210 | 7.1 | 19.44 | 8.9 |
| **Vines Creek** | 24/09/2019 14:11:53 | September 2019 | 27.74 | 7.21 | 46880 | 7.53 | 30.39 | 3.7 |
| **Vines Creek** | 22/11/2019 10:41:44 | November 2019 | 27.13 | 5.38 | 54140 | 7.39 | 35.74 | 6.25 |
| **Vines Creek** | 17/01/2020 10:06:44 | January 2020 | 29.76 | 4.45 | 42680 | 6.84 | 27.31 | 4.39 |
| **Vines Creek** | 18/03/2020 17:19:33 | March 2020 | 27.51 | 5.15 | 9200 | 7.08 | 5.13 | 6.94 |
| **Sandy Creek (Sandiford)** | 16/05/2019 16:25:56 | May 2019 | 22.67 | 6.69 | 640 | 7.63 | 0.31 | 20.4 |
| **Sandy Creek (Sandiford)** | 24/07/2019 9:06:34 | July 2019 | 19.09 | 7.46 | 1690 | 7.39 | 0.86 | 14.5 |
| **Sandy Creek (Sandiford)** | 25/09/2019 15:52:03 | September 2019 | 25.79 | 5.33 | 6890 | 7.56 | 3.77 | 26.1 |
| **Sandy Creek (Sandiford)** | 21/11/2019 17:05:21 | November 2019 | 30.36 | 10.33 | 7880 | 7.72 | 4.33 | 9.66 |
| **Sandy Creek (Sandiford)** | 15/01/2020 13:39:23 | January 2020 | 31.48 | 4.33 | 980 | 7.28 | 0.48 | 17.26 |
| **Sandy Creek (Sandiford)** | 20/03/2020 6:30:08 | March 2020 | 25.05 | 5.17 | 420 | 7.2 | 0.2 | 6 |
| **Sandy Creek (Eton)** | 16/05/2019 12:19:37 | May 2019 | 21.48 | 5.96 | 770 | 7.41 | 0.38 | 3.3 |
| **Sandy Creek (Eton)** | 24/07/2019 16:32:40 | July 2019 | 16.47 | 6.82 | 1420 | 7.73 | 0.72 | 1.2 |
| **Sandy Creek (Eton)** | 25/09/2019 16:36:40 | September 2019 | 21.59 | 8.37 | 1680 | 7.99 | 0.85 | 4.9 |
| **Sandy Creek (Eton)** | 21/11/2019 8:08:50 | November 2019 | 24.99 | 2.41 | 1500 | 7.57 | 0.75 | 2.09 |
| **Sandy Creek (Eton)** | 13/01/2020 18:04:53 | January 2020 | 27.38 | 1.24 | 660 | 7.09 | 0.32 | 0.3 |
| **Sandy Creek (Eton)** | 18/03/2020 16:19:19 | March 2020 | 24.69 | 5.54 | 470 | 7.43 | 0.23 | 2.63 |
| **Rocky Dam** | 13/05/2019 11:41:34 | May 2019 | 23.36 | 7.15 | 13000 | 7.89 | 7.48 | 133.5 |
| **Rocky Dam** | 24/07/2019 11:10:16 | July 2019 | 20.26 | 7.61 | 37810 | 7.88 | 24.05 | 18.3 |
| **Rocky Dam** | 25/09/2019 13:27:57 | September 2019 | 25.59 | 6.35 | 54330 | 7.98 | 35.93 | 35.9 |
| **Rocky Dam** | 21/11/2019 12:48:22 | November 2019 | 30.41 | 5.83 | 57740 | 7.66 | 38.33 | 29.39 |
| **Rocky Dam** | 15/01/2020 8:10:59 | January 2020 | 29.53 | 4.88 | 21230 | 7.54 | 12.65 | 159.35 |
| **Rocky Dam** | 19/03/2020 16:10:13 | March 2020 | 28.08 | 7.23 | 3230 | 7.92 | 1.68 | 135.03 |
| **Carmila Creek** | 13/05/2019 14:07:17 | May 2019 | 23.35 | 11.19 | 12610 | 8.23 | 7.24 | 28.8 |
| **Carmila Creek** | 24/07/2019 13:58:59 | July 2019 | 21.22 | 7.58 | 43930 | 7.82 | 28.39 | 21.4 |
| **Carmila Creek** | 25/09/2019 11:24:22 | September 2019 | 25.31 | 5.72 | 56060 | 7.85 | 37.22 | 33.5 |
| **Carmila Creek** | 21/11/2019 15:00:36 | November 2019 | 31.22 | 8.52 | 58730 | 7.63 | 39.04 | 16.66 |
| **Carmila Creek** | 15/01/2020 10:54:09 | January 2020 | 30.52 | 4.22 | 60330 | 7.27 | 40.28 | 20.87 |
| **Carmila Creek** | 19/03/2020 18:01:42 | March 2020 | 27.33 | 7.43 | 7220 | 7.76 | 3.95 | 20.79 |
| **Fitzroy River (Nerimbera)** | 22/05/2019 15:35:49 | May 2019 | 21.62 | 7.7 | 3730 | 8.07 | 1.97 | 53.2 |
| **Fitzroy River (Nerimbera)** | 18/07/2019 13:33:00 | July 2019 | 19.45 | 9.33 | 30310 | 8.31 | 18.85 | 30.3 |
| **Fitzroy River (Nerimbera)** | 18/09/2019 15:31:02 | September 2019 | 22.92 | 7.33 | 42480 | 7.82 | 27.33 | 7.5 |
| **Fitzroy River (Nerimbera)** | 27/11/2019 13:04:00 | November 2019 | 28.75 | 6.26 | 52690 | 7.95 | 34.62 | 3.6 |
| **Fitzroy River (Nerimbera)** | 14/01/2020 16:06:26 | January 2020 | 29.12 | 6.18 | 57300 | 7.61 | 38.05 | 8.9 |
| **Fitzroy River (Nerimbera)** | 25/03/2020 14:45:57 | March 2020 | 26.77 | 6.58 | 380 | 7.13 | 0.18 | 332 |
| **Fitzroy River (Barrage)** | 22/05/2019 10:33:15 | May 2019 | 21.94 | 7.29 | 250 | 7.54 | 0.12 | 215.5 |
| **Fitzroy River (Barrage)** | 18/07/2019 16:09:00 | July 2019 | 19.81 | 8.36 | 330 | 7.79 | 0.16 | 124.1 |
| **Fitzroy River (Barrage)** | 18/09/2019 11:00:09 | September 2019 | 21.51 | 7.83 | 250 | 7.34 | 0.12 | 91.7 |
| **Fitzroy River (Barrage)** | 27/11/2019 15:16:00 | November 2019 | 29.38 | 8.24 | 280 | 8.68 | 0.13 | 9.1 |
| **Fitzroy River (Barrage)** | 14/01/2020 11:37:52 | January 2020 | 29.4 | 8.14 | 400 | 8.35 | 0.19 | 5.6 |
| **Fitzroy River (Barrage)** | 25/03/2020 17:18:09 | March 2020 | 27.5 | 5.83 | 180 | 6.96 | 0.08 | 267.6 |
| **Calliope River** | 21/05/2019 16:44:34 | May 2019 | 23.03 | 6.31 | 53370 | 8.12 | 35.27 | 8.4 |
| **Calliope River** | 17/07/2019 16:11:00 | July 2019 | 18.85 | 7.44 | 55410 | 7.85 | 36.81 | 3.3 |
| **Calliope River** | 17/09/2019 15:28:03 | September 2019 | 22.69 | 6.76 | 57180 | 8 | 38.12 | 9.4 |
| **Calliope River** | 26/11/2019 13:45:00 | November 2019 | 29.23 | 5.57 | 59640 | 8.07 | 39.8 | 13.6 |
| **Calliope River** | 15/01/2020 17:22:56 | January 2020 | 30.12 | 5.59 | 60250 | 7.53 | 40.24 | 24.1 |
| **Calliope River** | 24/03/2020 15:33:02 | March 2020 | 28.38 | 7.94 | 33340 | 7.93 | 20.79 | 18 |
| **Auckland Creek** | 17/07/2019 14:26:00 | July 2019 | 19.35 | 7.25 | 55130 | 7.94 | 36.61 | 2.3 |
| **Auckland Creek** | 17/09/2019 16:26:30 | September 2019 | 22.61 | 7.22 | 55700 | 8.13 | 37.01 | 5.2 |
| **Auckland Creek** | 26/11/2019 11:59:00 | November 2019 | 27.59 | 6 | 57310 | 8.13 | 38.1 | 6.7 |
| **Auckland Creek** | 15/01/2020 11:45:42 | January 2020 | 28.39 | 5.97 | 57210 | 7.69 | 38 | 15.5 |
| **Auckland Creek** | 24/03/2020 13:14:11 | March 2020 | 26.41 | 6.38 | 52180 | 7.86 | 34.31 | 6.3 |
| **Boyne River** | 21/05/2019 13:58:19 | May 2019 | 22.82 | 7.31 | 48340 | 8.17 | 31.56 | 3.5 |
| **Boyne River** | 17/07/2019 11:41:00 | July 2019 | 18.56 | 7.3 | 47810 | 7.78 | 31.21 | 0.7 |
| **Boyne River** | 17/09/2019 12:34:04 | September 2019 | 23.01 | 6.84 | 51180 | 7.97 | 33.64 | 3.4 |
| **Boyne River** | 26/11/2019 10:10:00 | November 2019 | 28.7 | 5.34 | 55030 | 7.93 | 36.36 | 4.4 |
| **Boyne River** | 15/01/2020 14:08:34 | January 2020 | 29.99 | 5.81 | 56980 | 7.57 | 37.77 | 3.4 |
| **Boyne River** | 24/03/2020 11:32:45 | March 2020 | 27.96 | 6.22 | 33910 | 7.66 | 21.19 | 1.4 |
| **Baffle Creek** | 23/05/2019 13:46:06 | May 2019 | 23.25 | 6.95 | 45920 | 7.68 | 29.8 | 1.6 |
| **Baffle Creek** | 18/07/2019 12:25:22 | July 2019 | 17.23 | 7.5 | 47440 | 7.91 | 30.93 | 1.3 |
| **Baffle Creek** | 19/09/2019 15:44:00 | September 2019 | 25.01 | 6.93 | 50090 | 7.84 | 32.81 | 2.5 |
| **Baffle Creek** | 28/11/2019 13:00:58 | November 2019 | 30.04 | 6.64 | 56300 | 8.06 | 37.26 | 2.4 |
| **Baffle Creek** | 21/01/2020 9:08:32 | January 2020 | 30.69 | 5.68 | 55910 | 7.93 | 36.95 | 0.9 |
| **Baffle Creek** | 26/03/2020 14:55:33 | March 2020 | 27.62 | 6.39 | 3760 | 7.83 | 1.97 | 9.87 |
| **Burnett River (Fairymead)** | 23/05/2019 13:10:32 | May 2019 | 22.19 | 6.66 | 51750 | 8.15 | 34.08 | 2.7 |
| **Burnett River (Fairymead)** | 16/07/2019 11:14:00 | July 2019 | 17.83 | 7.09 | 51840 | 7.75 | 34.16 | 2.4 |
| **Burnett River (Fairymead)** | 19/09/2019 13:11:40 | September 2019 | 22.42 | 7.11 | 53270 | 7.99 | 35.2 | 2.9 |
| **Burnett River (Fairymead)** | 28/11/2019 11:32:00 | November 2019 | 27.7 | 6.25 | 53530 | 8.16 | 35.27 | 7 |
| **Burnett River (Fairymead)** | 16/01/2020 14:54:07 | January 2020 | 29.1 | 5.99 | 54940 | 7.77 | 36.28 | 4.8 |
| **Burnett River (Fairymead)** | 26/03/2020 13:12:10 | March 2020 | 26.25 | 6.94 | 47290 | 7.98 | 30.73 | 5.9 |
| **Burnett River (Bundaberg)** | 23/05/2019 13:47:18 | May 2019 | 22.71 | 7.26 | 47100 | 8.03 | 30.66 | 3.5 |
| **Burnett River (Bundaberg)** | 16/07/2019 11:57:00 | July 2019 | 18.9 | 6.95 | 46250 | 7.53 | 30.08 | 0.9 |
| **Burnett River (Bundaberg)** | 19/09/2019 13:59:49 | September 2019 | 23.35 | 6.51 | 49760 | 7.69 | 32.59 | 4.2 |
| **Burnett River (Bundaberg)** | 28/11/2019 12:10:00 | November 2019 | 28.35 | 6.3 | 46660 | 7.91 | 30.21 | 4.4 |
| **Burnett River (Bundaberg)** | 16/01/2020 15:33:28 | January 2020 | 28.98 | 6.04 | 51150 | 7.46 | 33.47 | 3.3 |
| **Burnett River (Bundaberg)** | 26/03/2020 14:18:59 | March 2020 | 26.87 | 10.64 | 37160 | 8.33 | 23.48 | 3 |
| **Burnett River (Oakwood)** | 23/05/2019 14:43:08 | May 2019 | 23.05 | 7.35 | 43290 | 8.06 | 27.9 | 9.2 |
| **Burnett River (Oakwood)** | 16/07/2019 12:45:00 | July 2019 | 18.47 | 8 | 43520 | 7.87 | 28.11 | 0.1 |
| **Burnett River (Oakwood)** | 19/09/2019 14:52:22 | September 2019 | 24.33 | 8.32 | 47400 | 7.98 | 30.85 | 9 |
| **Burnett River (Oakwood)** | 28/11/2019 13:02:00 | November 2019 | 28.75 | 5.81 | 42740 | 7.76 | 27.39 | 9.2 |
| **Burnett River (Oakwood)** | 16/01/2020 16:35:18 | January 2020 | 29.1 | 6.49 | 48070 | 7.46 | 31.21 | 6.9 |
| **Burnett River (Oakwood)** | 26/03/2020 15:12:29 | March 2020 | 26.65 | 10.11 | 26160 | 8.27 | 15.95 | 28.4 |
| **Burrum River** | 22/05/2019 14:31:12 | May 2019 | 22.41 | 7 | 41570 | 7.52 | 26.68 | 6.8 |
| **Burrum River** | 17/07/2019 13:42:11 | July 2019 | 17.44 | 9.61 | 46740 | 7.88 | 30.43 | 8.3 |
| **Burrum River** | 18/09/2019 15:02:00 | September 2019 | 22.64 | 5.85 | 52010 | 7.4 | 34.26 | 11.3 |
| **Burrum River** | 27/11/2019 12:58:14 | November 2019 | 29.05 | 7.68 | 55610 | 7.58 | 36.78 | 10.1 |
| **Burrum River** | 23/01/2020 12:33:38 | January 2020 | 30.88 | 5.03 | 55580 | 7.32 | 36.69 | 6.4 |
| **Burrum River** | 24/03/2020 13:35:41 | March 2020 | 26.95 | 7.01 | 9040 | 7.7 | 5.04 | 11.14 |
| **Isis River** | 22/05/2019 13:08:30 | May 2019 | 22.7 | 7.87 | 41990 | 7.69 | 26.98 | 20.3 |
| **Isis River** | 17/07/2019 11:53:43 | July 2019 | 15.72 | 7.33 | 47770 | 7.74 | 31.15 | 8.3 |
| **Isis River** | 18/09/2019 13:59:00 | September 2019 | 23.16 | 7.83 | 54510 | 7.72 | 36.11 | 15.4 |
| **Isis River** | 27/11/2019 11:45:01 | November 2019 | 28.13 | 4.71 | 62690 | 7.46 | 42.17 | 19.6 |
| **Isis River** | 23/01/2020 11:06:37 | January 2020 | 31.04 | 7.16 | 65330 | 7.89 | 44.09 | 8.5 |
| **Isis River** | 24/03/2020 12:10:47 | March 2020 | 27.12 | 6.92 | 16000 | 7.85 | 9.33 | 7.88 |
| **Gregory River (Woodgate)** | 22/05/2019 11:33:36 | May 2019 | 21.5 | 5.9 | 49300 | 7.58 | 32.29 | 1.1 |
| **Gregory River (Woodgate)** | 17/07/2019 10:09:43 | July 2019 | 17.11 | 6.83 | 52890 | 7.83 | 34.93 | 3 |
| **Gregory River (Woodgate)** | 18/09/2019 12:31:00 | September 2019 | 22.28 | 6.34 | 56700 | 7.85 | 37.76 | 4.5 |
| **Gregory River (Woodgate)** | 27/11/2019 10:22:03 | November 2019 | 27.54 | 5.49 | 58910 | 7.88 | 39.31 | 4.1 |
| **Gregory River (Woodgate)** | 23/01/2020 9:39:13 | January 2020 | 30.27 | 4.86 | 59120 | 7.87 | 39.38 | 5.1 |
| **Gregory River (Woodgate)** | 24/03/2020 10:16:41 | March 2020 | 26.71 | 4.85 | 33910 | 7.67 | 21.22 | 3.62 |
| **Mary River (Prawle)** | 15/05/2019 11:09:58 | May 2019 | 22.27 | 6.43 | 5540 | 7.51 | 3 | 13.2 |
| **Mary River (Prawle)** | 15/07/2019 13:55:00 | July 2019 | 18.78 | 7.64 | 5280 | 7.45 | 2.86 | 61.9 |
| **Mary River (Prawle)** | 17/09/2019 13:02:06 | September 2019 | 21.47 | 7.49 | 40960 | 7.8 | 26.26 | 10.5 |
| **Mary River (Prawle)** | 27/11/2019 13:11:44 | November 2019 | 27.57 | 6.1 | 48810 | 7.8 | 31.8 | 38.7 |
| **Mary River (Prawle)** | 13/01/2020 14:15:20 | January 2020 | 28.02 | 6.05 | 52250 | 7.82 | 34.32 | 14.4 |
| **Mary River (Prawle)** | 11/03/2020 12:58:24 | March 2020 | 26.58 | 6.42 | 12770 | 7.42 | 7.31 | 45.7 |
| **Mary River (Maryborough)** | 15/05/2019 11:53:21 | May 2019 | 22.11 | 6.38 | 280 | 7.23 | 0.13 | 11.5 |
| **Mary River (Maryborough)** | 15/07/2019 14:41:00 | July 2019 | 18.52 | 8.93 | 480 | 7.84 | 0.23 | 48.9 |
| **Mary River (Maryborough)** | 17/09/2019 13:43:04 | September 2019 | 22.25 | 8.8 | 20040 | 7.98 | 11.97 | 14.4 |
| **Mary River (Maryborough)** | 27/11/2019 14:15:30 | November 2019 | 28.49 | 5.71 | 30830 | 7.56 | 19.07 | 60.6 |
| **Mary River (Maryborough)** | 13/01/2020 15:36:46 | January 2020 | 29.07 | 5.87 | 38070 | 7.41 | 24.06 | 155.2 |
| **Mary River (Maryborough)** | 11/03/2020 13:51:56 | March 2020 | 27.52 | 5.58 | 590 | 7.31 | 0.28 | 162.1 |
| **Mary River (Grahams Creek)** | 15/05/2019 12:57:03 | May 2019 | 22.31 | 7.16 | 250 | 7.32 | 0.12 | 6.2 |
| **Mary River (Grahams Creek)** | 15/07/2019 15:55:00 | July 2019 | 17.91 | 8.74 | 310 | 7.26 | 0.15 | 7.9 |
| **Mary River (Grahams Creek)** | 17/09/2019 14:42:53 | September 2019 | 21.05 | 7.85 | 1060 | 7.94 | 0.52 | 20.1 |
| **Mary River (Grahams Creek)** | 27/11/2019 15:53:55 | November 2019 | 28.74 | 9.79 | 14190 | 8.21 | 8.17 | 9.2 |
| **Mary River (Grahams Creek)** | 13/01/2020 16:57:14 | January 2020 | 29.07 | 7.33 | 23630 | 7.64 | 14.23 | 9.5 |
| **Mary River (Grahams Creek)** | 11/03/2020 15:23:41 | March 2020 | 27.81 | 6.18 | 320 | 7.34 | 0.15 | 26.8 |
| **Great Sandy Straits (Northern end)** | 21/05/2019 15:15:03 | May 2019 | 22.17 | 7.02 | 54090 | 8.11 | 35.81 | 2 |
| **Great Sandy Straits (Northern end)** | 16/07/2019 13:15:09 | July 2019 | 18.07 | 7.48 | 53460 | 8.22 | 35.35 | 0.5 |
| **Great Sandy Straits (Northern end)** | 17/09/2019 14:24:00 | September 2019 | 21.02 | 7.53 | 54230 | 8.21 | 35.93 | 0.8 |
| **Great Sandy Straits (Northern end)** | 26/11/2019 14:09:42 | November 2019 | 26.91 | 6.71 | 55400 | 8.4 | 36.69 | 1.8 |
| **Great Sandy Straits (Northern end)** | 22/01/2020 12:18:02 | January 2020 | 28.79 | 6.27 | 55900 | 8.24 | 37 | 1.5 |
| **Great Sandy Straits (Northern end)** | 25/03/2020 14:41:03 | March 2020 | 25.94 | 6.67 | 54240 | 8.27 | 35.85 | 0.27 |
| **Great Sandy Straits (Southern end)** | 21/05/2019 11:16:02 | May 2019 | 21.34 | 7.02 | 52730 | 8.02 | 34.81 | 1 |
| **Great Sandy Straits (Southern end)** | 16/07/2019 10:19:31 | July 2019 | 16.78 | 7.54 | 53080 | 8.12 | 35.07 | 1.1 |
| **Great Sandy Straits (Southern end)** | 17/09/2019 11:16:00 | September 2019 | 21.4 | 7.29 | 54230 | 8.07 | 35.92 | 1.5 |
| **Great Sandy Straits (Southern end)** | 26/11/2019 11:17:45 | November 2019 | 26.98 | 6.49 | 55980 | 8.32 | 37.12 | 1.6 |
| **Great Sandy Straits (Southern end)** | 22/01/2020 10:01:11 | January 2020 | 29.38 | 6.05 | 56070 | 8.14 | 37.12 | 1.5 |
| **Great Sandy Straits (Southern end)** | 25/03/2020 9:47:30 | March 2020 | 25.61 | 6.37 | 50590 | 8.11 | 33.15 | 2.87 |
| **Noosa River (Noosaville)** | 17/05/2019 10:12:13 | May 2019 | 22.62 | 6.77 | 52810 | 8.2 | 34.86 | 0.4 |
| **Noosa River (Noosaville)** | 19/07/2019 13:15:22 | July 2019 | 18.22 | 7.63 | 47550 | 8.22 | 31.04 | 2.6 |
| **Noosa River (Noosaville)** | 18/09/2019 11:48:39 | September 2019 | 22.21 | 7.33 | 52860 | 8.03 | 34.9 | 1.1 |
| **Noosa River (Noosaville)** | 26/11/2019 12:44:54 | November 2019 | 26.18 | 6.61 | 53540 | 7.91 | 35.32 | 2.8 |
| **Noosa River (Noosaville)** | 20/01/2020 11:29:57 | January 2020 | 28.59 | 5.99 | 46680 | 7.98 | 30.22 | 2.4 |
| **Noosa River (Noosaville)** | 12/03/2020 13:05:00 | March 2020 | 25.81 | 6.7 | 50970 | 8.12 | 33.43 | 2.3 |
| **Noosa River (Cooroibah)** | 17/05/2019 11:21:02 | May 2019 | 20.48 | 6.92 | 31580 | 8 | 19.71 | 6.9 |
| **Noosa River (Cooroibah)** | 19/07/2019 12:07:18 | July 2019 | 16.07 | 8.37 | 21950 | 7.98 | 13.25 | 5.9 |
| **Noosa River (Cooroibah)** | 18/09/2019 12:49:24 | September 2019 | 22.96 | 7 | 30200 | 7.72 | 18.74 | 9.9 |
| **Noosa River (Cooroibah)** | 26/11/2019 14:03:36 | November 2019 | 27.48 | 6.15 | 47680 | 7.81 | 30.98 | 8.1 |
| **Noosa River (Cooroibah)** | 20/01/2020 12:29:48 | January 2020 | 28.66 | 5.95 | 37900 | 7.88 | 23.96 | 10.8 |
| **Noosa River (Cooroibah)** | 12/03/2020 14:22:00 | March 2020 | 24.67 | 6.85 | 13630 | 7.06 | 7.86 | 9.2 |
| **Noosa River (Lake Cootharaba)** | 17/05/2019 12:12:43 | May 2019 | 20.5 | 8.32 | 3860 | 7.53 | 2.05 | 5.6 |
| **Noosa River (Lake Cootharaba)** | 19/07/2019 11:39:15 | July 2019 | 16.34 | 9.56 | 7730 | 8.25 | 4.31 | 2.7 |
| **Noosa River (Lake Cootharaba)** | 18/09/2019 13:38:38 | September 2019 | 22.75 | 8.25 | 20080 | 7.86 | 12 | 2.8 |
| **Noosa River (Lake Cootharaba)** | 26/11/2019 15:13:36 | November 2019 | 27.77 | 7.27 | 32680 | 7.92 | 20.35 | 6.6 |
| **Noosa River (Lake Cootharaba)** | 20/01/2020 12:53:27 | January 2020 | 29.56 | 6.23 | 36390 | 7.96 | 22.88 | 7.8 |
| **Noosa River (Lake Cootharaba)** | 12/03/2020 15:41:00 | March 2020 | 24.37 | 7.55 | 500 | 6.44 | 0.24 | 22.6 |
| **Tin Can Inlet (Cooloola)** | 20/05/2019 14:24:12 | May 2019 | 22.2 | 7.45 | 34450 | 7.71 | 21.65 | 7.2 |
| **Tin Can Inlet (Cooloola)** | 15/07/2019 12:02:48 | July 2019 | 15.43 | 7.9 | 46200 | 7.91 | 30.01 | 5.1 |
| **Tin Can Inlet (Cooloola)** | 16/09/2019 13:31:00 | September 2019 | 22.48 | 7.22 | 54680 | 7.98 | 36.25 | 2.4 |
| **Tin Can Inlet (Cooloola)** | 25/11/2019 13:01:19 | November 2019 | 28.03 | 6.3 | 59790 | 8.2 | 39.96 | 9.2 |
| **Tin Can Inlet (Cooloola)** | 24/01/2020 11:26:19 | January 2020 | 28.4 | 6.08 | 53660 | 8.07 | 35.35 | 2.4 |
| **Tin Can Inlet (Cooloola)** | 23/03/2020 12:51:06 | March 2020 | 28.04 | 6.59 | 41300 | 7.96 | 26.38 | 4.72 |
| **Maroochy River** | 14/05/2019 10:42:43 | May 2019 | 20.71 | 5.38 | 6460 | 7.11 | 3.54 | 10.8 |
| **Maroochy River** | 19/07/2019 14:28:00 | July 2019 | 16.89 | 8.06 | 7540 | 7.43 | 4.19 | 12.2 |
| **Maroochy River** | 16/09/2019 12:27:30 | September 2019 | 21.38 | 7.89 | 33070 | 7.49 | 20.73 | 3 |
| **Maroochy River** | 28/11/2019 15:16:14 | November 2019 | 28.82 | 6.69 | 34080 | 7.51 | 21.29 | 10.6 |
| **Maroochy River** | 20/01/2020 10:05:06 | January 2020 | 27.98 | 3.23 | 26010 | 6.77 | 15.83 | 8.2 |
| **Maroochy River** | 10/03/2020 14:02:19 | March 2020 | 24.1 | 6.61 | 220 | 6.91 | 0.1 | 36.6 |
| **Bells Creek** | 16/05/2019 8:23:43 | May 2019 | 20.59 | 5.66 | 40580 | 7.59 | 26 | 3.6 |
| **Bells Creek** | 2/07/2019 9:57:17 | July 2019 | 17.55 | 5.95 | 2910 | 5.95 | 1.52 | 26.79 |
| **Bells Creek** | 2/09/2019 13:58:16 | September 2019 | 21.59 | 6.29 | 53310 | 7.71 | 35.24 | 5.3 |
| **Bells Creek** | 11/11/2019 10:12:32 | November 2019 | 26.01 | 5.27 | 54660 | 7.8 | 36.17 | 5.23 |
| **Bells Creek** | 14/01/2020 12:39:19 | January 2020 | 24.6 | 5.79 | 58080 | 7.74 | 38.76 | 5.9 |
| **Bells Creek** | 9/03/2020 11:00:23 | March 2020 | 24.96 | 5.59 | 28300 | 7.57 | 17.42 | 3.14 |
| **Pumicestone Passage** | 7/05/2019 15:12:30 | May 2019 | 22.26 | 7.65 | 44980 | 8 | 29.13 | 3.3 |
| **Pumicestone Passage** | 2/07/2019 12:42:30 | July 2019 | 19.55 | 7.41 | 33750 | 7.99 | 21.3 | 3.7 |
| **Pumicestone Passage** | 2/09/2019 15:37:33 | September 2019 | 21.48 | 7.54 | 55390 | 8.07 | 36.79 | 4.7 |
| **Pumicestone Passage** | 11/11/2019 12:00:01 | November 2019 | 26 | 6.72 | 58480 | 8.12 | 39.03 | 4.6 |
| **Pumicestone Passage** | 14/01/2020 13:55:24 | January 2020 | 24.87 | 5.97 | 58020 | 7.93 | 38.71 | 7.8 |
| **Pumicestone Passage** | 9/03/2020 12:08:30 | March 2020 | 24.91 | 5.68 | 35330 | 7.67 | 22.24 | 25.11 |
| **Coochin Creek** | **10/05/2019** | May 2019 | 18.6 | NR | 162 | NR | NR | NR |
| **Coochin Creek** | **11/07/2019** | July 2019 | 16.1 | NR | 152 | NR | NR | NR |
| **Coochin Creek** | **10/09/2019** | September 2019 | 15.3 | NR | 144 | NR | NR | NR |
| **Coochin Creek** | **19/11/2019** | November 2019 | 21.6 | NR | 135 | NR | NR | NR |
| **Coochin Creek** | **15/01/2020** | January 2020 | 25.2 | NR | 132 | NR | NR | NR |
| **Coochin Creek** | **17/03/2020** | March 2020 | 21.6 | NR | 163 | NR | NR | NR |
| **Caboolture River (Caboolture)** | 3/05/2019 10:39:31 | May 2019 | 21.91 | 8.13 | 210 | 7.46 | 0.1 | 8.4 |
| **Caboolture River (Caboolture)** | 2/07/2019 8:28:15 | July 2019 | 17.41 | 7.3 | 1590 | 7.35 | 0.81 | 3.45 |
| **Caboolture River (Caboolture)** | 2/09/2019 14:24:38 | September 2019 | 21.23 | 6.01 | 19460 | 7.32 | 11.61 | 2.86 |
| **Caboolture River (Caboolture)** | 8/11/2019 11:37:00 | November 2019 | 26.87 | 9.66 | 13660 | 7.66 | 7.86 | 6.8 |
| **Caboolture River (Caboolture)** | 9/01/2020 10:22:52 | January 2020 | 29.46 | 7.06 | 25110 | 7.52 | 15.2 | 8.3 |
| **Caboolture River (Caboolture)** | 9/03/2020 12:19:20 | March 2020 | 25.33 | 6.15 | 250 | 7.27 | 0.12 | 6.6 |
| **Caboolture River (Upper Caboolture)** | **10/05/2019** | May 2019 | 16.9 | NR | 341 | NR | NR | NR |
| **Caboolture River (Upper Caboolture)** | **11/07/2019** | July 2019 | 14.3 | NR | 356 | NR | NR | NR |
| **Caboolture River (Upper Caboolture)** | **10/09/2019** | September 2019 | 20.6 | NR | 757 | NR | NR | NR |
| **Brisbane River (Lytton)** | 1/05/2019 10:31:27 | May 2019 | 23.2 | 6.7 | 50550 | 8.1 | 33.18 | 3.4 |
| **Brisbane River (Lytton)** | 2/07/2019 11:34:15 | July 2019 | 18.05 | 7.6 | 45870 | 8.17 | 29.8 | 2.4 |
| **Brisbane River (Lytton)** | 9/09/2019 9:15:23 | September 2019 | 18.91 | 7.32 | 51110 | 8.07 | 33.62 | 2.01 |
| **Brisbane River (Lytton)** | 7/11/2019 10:17:57 | November 2019 | 24.3 | 6.77 | 51110 | 7.8 | 33.57 | 4.6 |
| **Brisbane River (Lytton)** | 9/01/2020 10:36:00 | January 2020 | 28.57 | 6.41 | 53040 | 8.02 | 34.88 | 4.1 |
| **Brisbane River (Lytton)** | 6/03/2020 11:31:18 | March 2020 | 28.09 | 5.84 | 43490 | 8.03 | 27.94 | 6.36 |
| **Brisbane River (Indooroopilly)** | 1/05/2019 12:40:32 | May 2019 | 24.31 | 6.11 | 20750 | 7.68 | 12.41 | 21.9 |
| **Brisbane River (Indooroopilly)** | 2/07/2019 13:17:31 | July 2019 | 18.8 | 7.32 | 20710 | 7.7 | 12.43 | 22.9 |
| **Brisbane River (Indooroopilly)** | 9/09/2019 11:23:38 | September 2019 | 19.32 | 7.62 | 25540 | 7.89 | 15.62 | 11.52 |
| **Brisbane River (Indooroopilly)** | 7/11/2019 12:10:24 | November 2019 | 25.38 | 6.61 | 26110 | 7.6 | 15.94 | 9.9 |
| **Brisbane River (Indooroopilly)** | 9/01/2020 13:15:25 | January 2020 | 29.65 | 5.67 | 26900 | 7.66 | 16.39 | 26.9 |
| **Brisbane River (Indooroopilly)** | 6/03/2020 13:35:29 | March 2020 | 28.66 | 3.46 | 8810 | 7.44 | 4.89 | 43.86 |
| **Brisbane River (Karana Downs)** | 1/05/2019 14:20:37 | May 2019 | 23.84 | 6.83 | 810 | 7.72 | 0.4 | 17.8 |
| **Brisbane River (Karana Downs)** | 2/07/2019 14:20:54 | July 2019 | 18.11 | 8.26 | 1506 | 7.95 | 0.76 | 7.1 |
| **Brisbane River (Karana Downs)** | 9/09/2019 13:57:34 | September 2019 | 19.68 | 8.92 | 2860 | 8.14 | 1.49 | 5.5 |
| **Brisbane River (Karana Downs)** | 7/11/2019 14:02:45 | November 2019 | 26.13 | 7.17 | 3180 | 7.85 | 1.66 | 8.9 |
| **Brisbane River (Karana Downs)** | 9/01/2020 18:20:09 | January 2020 | 29.19 | 7.04 | 3320 | 8.1 | 1.73 | 27.4 |
| **Brisbane River (Karana Downs)** | 6/03/2020 15:34:37 | March 2020 | 29.88 | 4.39 | 590 | 7.89 | 0.28 | 33.83 |
| **Oxley Creek** | 1/05/2019 10:30:10 | May 2019 | 24.16 | 6.09 | 21790 | 7.71 | 13.09 | 15.8 |
| **Oxley Creek** | 2/07/2019 13:02:13 | July 2019 | 18.2 | 6.96 | 16150 | 7.62 | 9.49 | 18.1 |
| **Oxley Creek** | 9/09/2019 9:54:23 | September 2019 | 18.94 | 7.33 | 25530 | 7.75 | 15.62 | 7.3 |
| **Oxley Creek** | 7/11/2019 9:01:00 | November 2019 | 24.64 | 6.5 | 27820 | 7.66 | 17.1 | 10.6 |
| **Oxley Creek** | 9/01/2020 12:38:37 | January 2020 | 29.64 | 5.37 | 27000 | 7.56 | 16.46 | 45 |
| **Oxley Creek** | 6/03/2020 9:41:22 | March 2020 | 28.32 | 3.75 | 13810 | 7.18 | 7.94 | 25.4 |
| **Warrill Creek** | 9/05/2019 | May 2019 | 17.1 | NR | 403 | NR | NR | NR |
| **Warrill Creek** | 7/07/2019 | July 2019 | 15 | NR | 454 | NR | NR | NR |
| **Warrill Creek** | 10/09/2019 | September 2019 | 14.2 | NR | 411 | NR | NR | NR |
| **Warrill Creek** | 22/11/2019 | November 2019 | 23.8 | NR | 433 | NR | NR | NR |
| **Warrill Creek** | 16/01/2020 | January 2020 | 24.9 | NR | 421 | NR | NR | NR |
| **Warrill Creek** | 25/03/2020 | March 2020 | 22.6 | NR | 714 | NR | NR | NR |
| **Logan River (Edens Landing)** | 8/05/2019 14:20:29 | May 2019 | 22.36 | 6.11 | 1870 | 7.54 | 0.95 | 153.5 |
| **Logan River (Edens Landing)** | 10/07/2019 9:33:32 | July 2019 | 17.8 | 7.72 | 2310 | 7.37 | 1.19 | 53.18 |
| **Logan River (Edens Landing)** | 10/09/2019 12:25:28 | September 2019 | 19.38 | 7.76 | 17410 | 7.77 | 10.29 | 11.96 |
| **Logan River (Edens Landing)** | 11/11/2019 13:26:46 | November 2019 | 26.12 | 5.49 | 27330 | 7.57 | 16.75 | 32.02 |
| **Logan River (Edens Landing)** | 7/01/2020 13:51:00 | January 2020 | 30.03 | 4.91 | 22840 | 7.44 | 13.69 | 48.2 |
| **Logan River (Edens Landing)** | 10/03/2020 13:26:25 | March 2020 | 26.34 | 5.16 | 550 | 7.61 | 0.26 | 40.18 |
| **Tingalpa Creek** | 7/05/2019 14:14:31 | May 2019 | 20.35 | 4.8 | 8000 | 7.31 | 4.45 | 3.87 |
| **Tingalpa Creek** | 10/07/2019 8:33:44 | July 2019 | 16.99 | 3.71 | 5840 | 6.99 | 3.19 | 3.7 |
| **Tingalpa Creek** | 11/09/2019 8:58:49 | September 2019 | 17.45 | 4.46 | 21290 | 7.3 | 12.82 | 4 |
| **Tingalpa Creek** | 8/11/2019 9:02:49 | November 2019 | 23.97 | 2.37 | 15750 | 7.14 | 9.2 | 2.76 |
| **Tingalpa Creek** | 8/01/2020 10:21:00 | January 2020 | 27.69 | 2.27 | 21480 | 7.08 | 12.83 | 6.7 |
| **Tingalpa Creek** | 9/03/2020 12:25:51 | March 2020 | 26.04 | 2.98 | 6890 | 6.81 | 3.77 | 6.09 |
| **Logan River (Glenlogan)** | 9/05/2019 | May 2019 | 20.3 | NR | 261 | NR | NR | NR |
| **Logan River (Glenlogan)** | 7/07/2019 | July 2019 | 17.3 | NR | 530 | NR | NR | NR |
| **Logan River (Glenlogan)** | 10/09/2019 | September 2019 | 15.4 | NR | 1128 | NR | NR | NR |
| **Logan River (Glenlogan)** | 16/01/2020 | January 2020 | 25.6 | NR | 1342 | NR | NR | NR |
| **Logan River (Glenlogan)** | 25/03/2020 | March 2020 | 25 | NR | 490 | NR | NR | NR |
| **Northern Broadwater (Couran Cove)** | 9/05/2019 14:12:18 | May 2019 | 22.15 | 6.95 | 52160 | 8.01 | 34.38 | 5.4 |
| **Northern Broadwater (Couran Cove)** | 10/09/2019 11:24:59 | September 2019 | 17.47 | 7.25 | 54720 | 8.08 | 36.29 | 5.3 |
| **Northern Broadwater (Couran Cove)** | 12/11/2019 13:06:00 | November 2019 | 24.43 | 6.83 | 54900 | 7.97 | 36.38 | 6.5 |
| **Northern Broadwater (Couran Cove)** | 29/01/2020 14:14:19 | January 2020 | 29.61 | 7.16 | 50430 | 8.18 | 32.92 | 10.8 |
| **Northern Broadwater (Couran Cove)** | 11/03/2020 13:56:16 | March 2020 | 24.21 | 6.85 | 43520 | 8.02 | 28.05 | 6.81 |
| **Northern Broadwater (Southport)** | 10/05/2019 13:53:29 | May 2019 | 23.66 | 6.91 | 51440 | 8.05 | 33.82 | 0.98 |
| **Northern Broadwater (Southport)** | 3/09/2019 13:30:16 | September 2019 | 19.84 | 7.43 | 53390 | 8.1 | 35.31 | 0.4 |
| **Northern Broadwater (Southport)** | 12/11/2019 11:48:13 | November 2019 | 22.8 | 7.27 | 54020 | 8.23 | 35.75 | 1.3 |
| **Northern Broadwater (Southport)** | 29/01/2020 14:46:52 | January 2020 | 27.24 | 7.63 | 49050 | 8.32 | 31.99 | 2.1 |
| **Northern Broadwater (Southport)** | 12/03/2020 13:55:11 | March 2020 | 25.01 | 6.88 | 47830 | 8.03 | 31.15 | 2.6 |
| **Moreton Bay (N of Russell Island)** | 8/05/2019 14:21:42 | May 2019 | 22.33 | 7.03 | 53610 | 8.16 | 35.45 | 1.15 |
| **Moreton Bay (N of Russell Island)** | 8/07/2019 16:22:35 | July 2019 | 18.57 | 8.15 | 47100 | 8.21 | 30.69 | 1.34 |
| **Moreton Bay (N of Russell Island)** | 4/09/2019 13:36:35 | September 2019 | 20.34 | 7.76 | 54030 | 8.16 | 35.78 | 0.79 |
| **Moreton Bay (N of Russell Island)** | 14/11/2019 14:11:28 | November 2019 | 25.16 | 7.36 | 55290 | 8.25 | 36.66 | 3.18 |
| **Moreton Bay (N of Russell Island)** | 8/01/2020 14:29:42 | January 2020 | 29.14 | 6.95 | 56270 | 7.99 | 37.27 | 8.7 |
| **Moreton Bay (N of Russell Island)** | 19/03/2020 10:13:33 | March 2020 | 23.24 | 6.93 | 49790 | 7.95 | 32.62 | 3.8 |
| **Moreton Bay (Near Moreton Island)** | 13/05/2019 9:02:36 | May 2019 | 22.25 | 6.83 | 53840 | 8.28 | 35.63 | 0.1 |
| **Moreton Bay (Near Moreton Island)** | 9/07/2019 14:59:16 | July 2019 | 19.15 | 7.74 | 48610 | 8.21 | 31.79 | 2.1 |
| **Moreton Bay (Near Moreton Island)** | 9/09/2019 10:32:28 | September 2019 | 19.22 | 7.46 | 53750 | 8.07 | 35.58 | 0.6 |
| **Moreton Bay (Near Moreton Island)** | 7/11/2019 11:13:43 | November 2019 | 23.5 | 7.13 | 54060 | 8.23 | 35.77 | 0.04 |
| **Moreton Bay (Near Moreton Island)** | 20/03/2020 9:26:17 | March 2020 | 24.3 | 7.02 | 52230 | 7.99 | 34.4 | 0.1 |
| **Moreton Bay (Bramble Bay)** | 13/05/2019 10:42:36 | May 2019 | 19.33 | 7.02 | 52010 | 8.24 | 34.29 | 4.2 |
| **Moreton Bay (Bramble Bay)** | 9/07/2019 15:45:00 | July 2019 | 18.35 | 8.15 | 47830 | 8.2 | 31.22 | 2.9 |
| **Moreton Bay (Bramble Bay)** | 9/09/2019 12:29:33 | September 2019 | 17.53 | 7.83 | 54360 | 8.02 | 36.02 | 1.6 |
| **Moreton Bay (Bramble Bay)** | 7/11/2019 12:33:01 | November 2019 | 23.92 | 7.15 | 55020 | 8.22 | 36.48 | 1.11 |
| **Moreton Bay (Bramble Bay)** | 21/01/2020 11:00:23 | January 2020 | 28.33 | 6.59 | 51640 | 7.94 | 33.86 | 3.5 |
| **Moreton Bay (Bramble Bay)** | 11/03/2020 13:52:01 | March 2020 | 25.06 | 7.26 | 47510 | 8.28 | 30.92 | 8.69 |

**Table S3** PFAS and reporting limits

| PFAS groups and chemical names | PFAS acronym | CAS number | LOR (ng/L) |
| --- | --- | --- | --- |
| **Perfluoroalkyl carboxylic acids (PFCAs)** | | | |
| Perfluoropentanoic acid | PFBA | 375-22-4 | 5 |
| Perfluoropentanoic acid | PFPeA | 2706-90-3 | 1 |
| Perfluorohexanoic acid | PFHxA | 307-24-4 | 1 |
| Perfluoroheptanoic acid | PFHpA | 375-85-9 | 1 |
| Perfluorooctanoic acid | PFOA | 335-67-1 | 1 |
| Perfluorononanoic acid | PFNA | 375-95-1 | 1 |
| Perfluorodecanoic acid | PFDA | 335-76-2 | 1 |
| Perfluoroundecanoic acid | PFUnDA | 2058-94-8 | 1 |
| Perfluorododecanoic acid | PFDoDA | 307-55-1 | 1 |
| Perfluorotridecanoic acid | PFTrDA | 72629-94-8 | 1 |
| Perfluorotetradecanoic acid | PFTeDA | 376-06-7 | 1 |
| **Perfluoroalkyl sulfonic acids (PFSAs)** | | | |
| Perfluoropropanesulfonic acid | PFPrS | 423-41-6 | 1 |
| Perfluorobutanesulfonic acid | PFBS | 375-73-5 | 1 |
| Perfluoropentanesulfonic acid | PFPeS | 2706-91-4 | 1 |
| Perfluorohexanesulfonic acid | PFHxS | 335-46-4 | 1 |
| Perfluoroheptanesulfonic acid | PFHpS | 375-92-8 | 1 |
| Perfluorooctanesulfonic acid | PFOS | 1763-23-1 | 0.1 |
| Perfluorononanesulfonic acid | PFNS | 68259-12-1 | 1 |
| Perfluorodecanesulfonic acid | PFDS | 335-77-3 | 1 |
| **Fluorotelomer sulfonic acids** **(FTSAs)** | | | |
| 1H.1H.2H.2H-perfluorohexanesulfonic acid | 4:2 FTSA | 757124-72-4 | 1 |
| 1H.1H.2H.2H-perfluorooctanesulfonic acid | 6:2 FTSA | 27619-97-2 | 5 |
| 1H.1H.2H.2H-perfluorodecanesulfonic acid | 8:2 FTSA | 39108-34-4 | 1 |
| 1H.1H.2H.2H-perfluorododecanesulfonic acid | 10:2 FTSA | 120226-60-0 | 1 |
| **Perfluoroalkyl sulfonamido substances (FASAs)** | | | |
| 2-(N-ethylperfluoro-1-octane sulfonamido)-ethanol | N-EtFOSE | 1691-99-2 | 5 |
| 2-(N-methylperfluoro-1-octane sulfonamido)-ethanol | N-MeFOSE | 24448-09-7 | 5 |
| N-ethylperfluoro-1-octane sulfonamide | N-EtFOSA | 4151-50-2 | 5 |
| N-ethyl-perfluorooctanesulfonamidoacetic acid | N-EtFOSAA | 2991-50-6 | 5 |
| N-methylperfluoro-1-octane sulfonamide | N-MeFOSA | 31506-32-8 | 5 |
| N-methyl-perfluorooctanesulfonamidoacetic acid | N-MeFOSAA | 2355-31-9 | 5 |
| Perfluorooctane sulfonamide | FOSA | 754-91-6 | 5 |

Quality Control

Overview

A summary of the quality control (QC) samples that were collected for each sampling round are presented Table S4.

**Table S4** Summary of number of QC samples taken for each sampling round

| Monitoring round | Field Blank | Trip Blank | Duplicates (intra-lab) | Triplicates (inter-lab) |
| --- | --- | --- | --- | --- |
| May | 7 | 7 | 6 |  |
| July | 6 | 6 | 8 |  |
| September^1^ | 6 | 5 | 8 |  |
| November^2^ | 7 | 7 | 6 | 3 |
| January | 6 | 6 | 9 | 3 |
| March | 6 | 6 | 9 | 4 |

^1^Quality control results from the September 2019 indicated internal quality issues. Samples were re-analysed by the laboratory after these issues had been addressed. Only the results of the second analysis are presented

^2^The November monitoring round were analysed twice by the primary laboratory as an additional QC measure due to the issues that occurred in the September monitoring round

Field and trip blanks

The reported concentrations of PFAS in transport blanks were below the LOR in all instances. The reported concentrations of PFAS in field blanks collected in May, July and September 2019, and March 2020 were below the LOR. In November 2019 the reported concentration of PFOS in a field blank collected at Rocky Dam was 35 ng L^-1^ (Table S5). The sample was analysed a second time, and a concentration of 24 ng L^-1^ was reported. The cause of the high concentration in the field blank is not known. However, the environmental sample collected at the same site had a reported PFOS concentration below the LOR (0.1 ng L^-1^), indicating the contamination was either not widespread or the positive result was due to laboratory error. A second field blank collected in November 2019 appeared to have contamination. PFTrDA was reported at 2 ng L^-1^ (LOR of 1 ng L^-1^) at Warrill Creek, however no PFTrDA was reported above the LOR when the sample was analysed a second time, and no PFTrDA was reported above the LOR in the environmental sample collected at this site. This field blank result was treated as an outlier.

In January 2020 low concentrations of PFOS (0.2 ng L^-1^, LOR 0.1 ng L^-1^) were reported in the field blank collected at Tin Can Inlet, however the reported PFOS concentration was not higher than the LOR in the environmental sample collected at this site, indicating the result may be due to laboratory error. In the same month, the reported concentration of PFOS (0.2 ng L^-1^, LOR 0.1 µg/L), in the field blank collected at the Burnett River site was lower than the concentration reported in the environmental sample collected at this site (0.4 ng L^-1^). Results for PFOS concentrations at 0.2 ng L^-1^ or lower collected by this field team in January 2020 were adjusted to account for the potential contamination (subtracted from reported concentrations).

Duplicates (intra-laboratory)

For intra-laboratory (intra-lab) duplicates, the relative percentage difference (RPD) was calculated. Allowable RPD limits were set for the following ranges:

- reported concentration 1–10 times the LOR: 100%
- reported concentration greater than 10 times the LOR: 30%.

The RPDs were within acceptance criteria for all intra-lab duplicate samples collected in May, July and November 2019, as well as March 2020.

In September 2019, QC results indicated internal laboratory quality issues. Samples were re-analysed by the laboratory after these issues had been addressed and the remaining RPDs exceedances are summarised below (Table S6). These should be interpreted with caution.

In January 2020, the duplicates samples collected at Tingalpa Creek had a RPD for PFOS of 120%, the reported results however were between 4 ng L^-1^ and the LOR of 1 ng L^-1^ (Table S6).

**Table S5** Summary of data for field blanks where PFAS concentrations were reported >LOR. All concentrations are ng L^-1^

| Monitoring round | Location | Compound | PFOS | PFTrDA |
| --- | --- | --- | --- | --- |
|  |  | LOR | 0.1 | 1 |
| November | Warrill Creek | Run 1 | <0.1 | **2** |
|  |  | Run 2 | <0.1 | <1 |
|  | Rocky Dam Creek | Run 1 | **35** | <1 |
|  |  | Run 2 | **24** | <1 |
| January | Tin Can Inlet | – | **0.2** | <1 |
|  | Burnett River (Bundaberg) | – | **0.2** | <1 |

**Table S6** Summary of data for duplicates (intra-lab) where PFAS concentrations were reported >LOR. All concentrations are ng L^-1^

| Monitoring round | Location | Compound | PFOS | PFHxS | PFBS |
| --- | --- | --- | --- | --- | --- |
|  |  | **LOR** | **0.1** | **1** | **1** |
| September | Logan River (Edens Landing) | | **3.1** |  | |
|  |  |  | **1.6** |  |  |
| **RPD %** | | | **64** |  |  |
| September | Oxley Creek | | **12** | **11** | 4 |
|  |  |  | **8.7** | **8** | 1 |
| **RPD %** | | | **32** | **32** | 120 |
| January | Tingalpa Creek | | **0.4** |  | |
|  |  |  | <0.1 |  |  |
| **RPD %** | | | 120 |  |  |

Duplicates (inter-laboratory)

Three inter-lab duplicates were analysed each in November 2019 and January 2020 and four in March 2020. For the inter-lab duplicates, the RPD was calculated and compared against the same acceptable criteria as the intra-lab duplicates. Where the LOR of the primary and secondary laboratories differed, the more conservative LOR was used to set the acceptance criteria. No RPD was calculated if one of the laboratories reported an analyte below the LOR.

The following PFAS had exceedances of RPD ranges for inter-lab samples, however passed the intra-lab QC (Table S7):

- Perfluorohexane sulfonic acid (PFHxS) in one sample in November 2019 (42%) and two samples in March 2020 (47% and 35%); the results of these samples were within 10–30 times the LOR
- Perfluorobutane sulfonic acid (PFBS) for two samples collected in January 2020 (107% and 111%); the results of these samples were within 1–10 times the LOR

Results were rerun and confirmed, however it was acknowledged that there is no standard method for PFAS analyses and method uncertainty may cause some variability in results reported (particularly between labs).

**Table S7** Summary of data for duplicates (inter-lab) where PFAS concentrations were reported >LOR. All concentrations are ng L^-1^

| Monitoring round | Location | | Compound | PFHxS | PFBS |
| --- | --- | --- | --- | --- | --- |
|  |  |  | LOR (Lab 1) | 1 | 1 |
|  |  |  | LOR (Lab 2) | 0.5 | 0.5 |
| November | Brisbane River (Karana Downs) | Lab 1  Lab 1  Lab 2 | | **12** | 3 |
|  |  |  |  | **12** | 2 |
|  |  |  |  | **18.4** | <0.5 |
|  | **RPD (%)** | | | **42** | NA |
| January | Brisbane River (Indooroopilly) | Lab 1  Lab 1  Lab 2 | | 7 | **2** |
|  |  |  |  | 8 | **2** |
|  |  |  |  | 8.3 | **6.6** |
|  | **RPD (%)** | | | 4 | **107** |
|  | Oxley Creek | Lab 1  Lab 1  Lab 2 | | 8 | **2** |
|  |  |  |  | 8 | **2** |
|  |  |  |  | 8.8 | **7** |
|  | **RPD (%)** | | | 10 | **111** |
|  | Brisbane River (Indooroopilly) | Lab 1  Lab 1  Lab 2 | | **16** | 3 |
| March |  |  |  | **18** | 3 |
|  |  |  |  | **29.1** | 5.4 |
|  | **RPD (%)** | | | **47** | 57 |
|  | Oxley Creek | Lab 1  Lab 1  Lab 2 | | **15** | 3 |
|  |  |  |  | **17** | 3 |
|  |  |  |  | **24.3** | 4.3 |
|  | **RPD (%)** | | | **35** | 36 |

Spatial analysis methods

First, we obtained two datasets through the Google Earth Engine platform: the **WWF HydroSHEDS level 6 and level 12 Basin (Lehner and Grill, 2013)** datasets (in .shp format), which provides hydrologically consistent watershed boundaries at a global scale, and the **CIESIN Gridded Population of the World (GPWv4.11) Population Density for 2020 (CIESIN, 2018)** dataset (in .tiff format), which offers spatially explicit population density estimates at approximately 1km (30 arc-seconds) resolution. The average size of level 12 basins was 180 km² and the average size of level 6 basins was 12 050 km^2^. Both datasets were filtered to the geographical extent of Queensland, Australia. After exporting both datasets, the **zonal statistics** tool in QGIS (version 3.38.2) was employed to calculate the sum of population per basin with the cell count representing the basin size. This approach allowed for a spatially explicit assessment of population distribution across the various hydrological basins in Queensland. The PFAS site latitude and longitude was then used to join the PFAS concentrations to the respective basin’s population data.

To further investigate the potential influence of runoff on PFAS concentrations at each sampling site, we assign a relative elevation to each site within its respective catchment area. Recognizing that sites at higher elevations within the same basin might experience different runoff dynamics, and therefore potentially different PFAS loads, we aimed to quantify the relative position of each site to account for these variations.

We used the **Shuttle Radar Topography Mission (SRTM) elevation data**, accessed via the **AusMap QGIS plugin** ([WMS, [29/08/2024]](https://wmseng.com.au/)). Using a similar approach as described for the population analysis, we first derived the minimum and maximum elevation values for each basin using the zonal statistics tool in QGIS (version 3.38.2).

Next, we sampled the SRTM elevation data at each sampling location to obtain the specific elevation of each site. To express the relative elevation of each site within its respective basin, we performed a **min-max normalisation**.

$$Normalised Elevation= \frac{(Elevation at Site-Minimum Elevation in Basin)}{(Maximum Elevation in Basin-Minimum Elevation in Basin)}$$

This normalisation process produced a dimensionless value ranging from 0 to 1, representing the relative position of each site within its catchment/basin. A value of 0 indicates the lowest elevation within the basin, while a value of 1 represents the highest point. Sites with intermediate values are positioned proportionately within the elevation range of their respective basins. The Hydrosheds level 12 and level 6 population data, as well as the normalised site elevation at both level 6 and level 12 were then included as additional variables for subsequent analysis (Fig. S2).

**Fig. S2** An illustration of WWF HydroSHEDS level 6 and level 12 hydrosheds, overlayed onto SRTM elevation data with sample site PFOS concentrations shaded in red.

|  |  |
| --- | --- |

**Fig. S3** Seasonal patterns in total PFAS at (a) Caboolture River (Caboolture) and (b) Brisbane River (Indooroopilly)

2. Water type as described in the Environmental Protection (Water and Wetland Biodiversity) Policy 2019.
3. The Australian Statistical Geography Standard (ASGS) Remoteness Structure according to the Australian Bureau of Statistics (ABS) (ABS, 2021)
